# Keratin 17 covalently binds to alpha-enolase and exacerbates proliferation of keratinocytes in psoriasis

**DOI:** 10.7150/ijbs.83141

**Published:** 2023-07-03

**Authors:** Yixin Luo, Bingyu Pang, Junfeng Hao, Qingyang Li, Pei Qiao, Chen Zhang, Yaxing Bai, Chunying Xiao, Jiaoling Chen, Dalong Zhi, Ying Liu, Erle Dang, Gang Wang, Bing Li

**Affiliations:** Department of Dermatology, Xijing Hospital, Fourth Military Medical University, Xi'an, Shaanxi 710032, China.

**Keywords:** Psoriasis, Keratin 17, Alpha-enolase, Glycolysis, Keratinocyte proliferation

## Abstract

Dysregulated glucose metabolism is an important characteristic of psoriasis. Cytoskeletal protein keratin 17 (K17) is highly expressed in the psoriatic epidermis and contributes to psoriasis pathogenesis. However, whether K17 is involved in the dysregulated glucose metabolism of keratinocytes (KCs) in psoriasis remains unclear. In the present study, loss- and gain-of-function studies showed that elevated K17 expression was critically involved in glycolytic pathway activation in psoriatic KCs. The level of α-enolase (ENO1), a novel potent interaction partner of K17, was also elevated in psoriatic KCs. Knockdown of ENO1 by siRNA or inhibition of ENO1 activity by the inhibitor ENOBlock remarkably suppressed KCs glycolysis and proliferation. Moreover, ENO1 directly interacted with K17 and maintained K17-Ser^44^ phosphorylation to promote the nuclear translocation of K17, which promoted the transcription of the key glycolysis enzyme lactic dehydrogenase A (*LDHA*) and resulted in enhanced KCs glycolysis and proliferation* in vitro*. Finally, either inhibiting the expression and activation of ENO1 or repressing K17-Ser^44^ phosphorylation significantly alleviated the IMQ-induced psoriasis-like phenotype *in vivo*. These findings provide new insights into the metabolic profile of psoriatic KCs and suggest that modulation of the ENO1-K17-LDHA axis is a potentially innovative therapeutic approach to psoriasis.

## Introduction

Psoriasis is a chronic relapsing autoimmune skin disease that is characterized by excessive proliferation of the epidermis, dilation of dermal capillaries and inflammatory cell infiltration 1-4. In these complex processes, the mechanism leading to hyperproliferation of epidermal keratinocytes (KCs) is of crucial importance, but there are still many gaps in our understanding.

Keratin 17 (K17) is a member of the keratin family and functions as a cytoskeletal protein 5-7. In the past two decades, our groups have found that K17 is abnormally expressed in epidermal KCs in psoriasis 8, 9. This elevated K17 expression upregulates multiple proinflammatory cytokines and chemokines produced by KCs, eventually forming a K17-T-cell-cytokine autoimmune loop that participates in the development of psoriasis 10-12. Additionally, recent studies found that K17 translocates into the nucleus and may function as a transcription factor to regulate the proliferation of KCs 13, 14. However, whether this process contributes to the pathogenesis of psoriasis and the underlying molecular mechanism remain unclear.

Glycolysis is an intracellular metabolic process and is necessary for cell proliferation 15, 16. The pathway consists of 10 consecutive enzymatic reactions that catabolize 1 molecule of glucose into 2 molecules of pyruvate 17, 18. Disorders in glucose metabolism of cells have been shown to be involved in skin tumorigenesis 19, 20. Recent studies have shown that increased glycolysis promotes glucose uptake in KCs and promotes cell proliferation, suggesting a major role of glycolysis in KCs fate 21. Moreover, emerging evidence illustrates that lactic acid, a byproduct of glycolysis generated from pyruvate by lactate dehydrogenase (LDH) as part of aerobic glycolysis, has been reported to be markedly increased in psoriatic lesional skin 22, 23. In addition, anaerobic glycolysis induced by upregulation of PPARδ is an advantageous metabolic pathway to sustain forced KCs proliferation because it is a substantial source of ATP 24. These studies suggest that glycolysis may be involved in the pathogenesis of psoriasis by regulating keratinocyte proliferation, yet the specific molecular mechanisms are unclear.

α-Enolase (ENO1), also known as 2-phospho-D-glycerate hydrolase, catalyzes the conversion of 2-phosphoglyceric acid to phosphoenolpyruvic acid during the penultimate step in glycolysis 25, 26. The expression of ENO1 can be activated by several glucose transporters and glycolytic enzymes that participate in tumor development 27. Moreover, overexpression of ENO1, which is regulated by multiple mechanisms, can promote tumor growth and development in a myriad of cancer types 28-30. Furthermore, the downregulation of ENO1 protein in non-small lung cancer cells indicates a tumor suppressor function of ENO1, indicating that ENO1 plays an important role in cell proliferation 31, 32. However, the physiological and pathological roles of ENO1 in psoriasis have not yet been fully elucidated, and whether ENO1 and K17 act together to contribute to abnormal glycolysis and excessive proliferation of KCs in psoriasis has not previously been examined.

This study aimed to evaluate the relationship between ENO1 and K17 in promoting the development of psoriasis. We identified ENO1 as a novel potent interaction partner of K17. ENO1 expression was significantly increased in psoriatic KCs and induced by elevated K17. Furthermore, we confirmed that ENO1 could covalently interact with K17 and prompt the phosphorylation of K17-Ser^44^ to promote nuclear translocation of K17, which increases the transcription of LDHA to contribute to glycolysis and proliferation of KCs. This further induces abnormal glycolysis and proliferation of KCs, thereby accelerating the process of psoriasis development. These results provide novel insights into the potential role of K17 and ENO1 in the pathogenesis of psoriasis.

## Materials and methods

### Human subjects

All clinical tissue samples from patients with psoriasis were obtained from populations in China and were collected at the Department of Dermatology, Xijing Hospital. The research was approved by the local medical research ethics committee at Xijing Hospital, the Fourth Military Medical University, Xi'an, China (Ethics Committee approval No: KY20203160-1). Informed consent regarding the use of specimens was obtained from all patients.

### Experimental animals

Prof. Pierre A. Coulombe (University of Michigan, Ann Arbor, MI, USA) kindly donated K17 knockout mice with a C57BL/6J genetic background. Female C57BL/6J mice were purchased from the Fourth Military Medical University's Laboratory Animal Medicine Department when they were 8-10 weeks old. All animal experiments were performed according to the animal experimental protocols under ethical approval by the Fourth Military Medical University (Xi'an, China).

To establish the IMQ-induced psoriasis model in the mouse ears, the right ear received a topical dose of 20 mg IMQ (5% IMQ, INova Pharmaceuticals, Chatswood, NSW, Australia) for 6 consecutive days, whereas the control mice received the same dose of vehicle (Vaseline; Unilever, Englewood Cliffs, USA). To evaluate the effect of ENO1 inhibition or phosphorylation of K17-Ser^44^ inhibition on the development of psoriasis, 2.5 nmol/kg ENO1 siRNA (Ribobio, Guangzhou, China), 10 mg/kg ENOBlock (Ribobio, Guangzhou, China), and 0.5 mg/kg BI-D1870 (Ribobio, Guangzhou, China) were individually mixed with vehicle and applied topically to the right ear of mice after 3-4 h of challenge with IMQ every other day for 5 days. Control group mice were treated with 2.5 nmol/kg negative control siRNA and the same volume of DMSO. The ear thickness was measured using an engineer's micrometer caliper at the indicated times. The ear samples were fixed and prepared for further histological analysis.

### Preparation, culture and treatment of human primary KCs

Human primary KCs were collected from the foreskins of twelve patients (aged 8 to 30 years) who underwent urological surgery at the Department of Urology, Xijing Hospital, as previously described 33. Keratinocyte complete media (EpiLife, Thermo Fisher Scientific, USA) supplemented with EpiLife medium +60 µM calcium/EpiLife Defined Growth Supplement (EDGS, Thermo Fisher Scientific, USA) was used to sustain human primary KCs. Penicillin‒streptomycin (50 U/mL) was added. Human primary KCs were then treated with ENO1 siRNA, ENOBlock, BI-D1870, K17 and mutant K17 overexpression plasmids in 6-well plates.

The human HaCaT keratinocyte cell line was purchased from KeyGEN Biotech (GB300, Nanjing, China), cultured in Dulbecco's Modified Eagle's medium (DMEM; Gibco-Invitrogen, Carlsbad, USA) supplemented with 10% fetal bovine serum (FBS; Gibco-Invitrogen, Carlsbad, USA) and maintained at 37°C in a humidified environment with 5% CO_2_. After 24 h of starvation, cells at 40-60% confluence were stimulated with IL-17, IL-22, tumor necrosis factor-α (TNF-α), IL-1α and oncostatin M (OSM) (50, 20, 50, 20 and 20 ng/mL, respectively) (PeproTech, Rocky Hill, NJ, USA) for 48 h, and PBS treatment served as a negative control.

### Extracellular acidification rate (ECAR) test

The ECAR was evaluated using a Seahorse XF Glycolysis Stress Test Kit and measured by an XF96 Extracellular Flux Analyzer (Agilent, Santa Clara, USA) in accordance with the manufacturer's instructions as previously mentioned 34. Briefly, 5-6×10^3^ cells were then plated into Seahorse XF96 cell culture microplates for 24 h. Before measurement, cells were incubated in a non-CO_2_ injected incubator for 1 h to rule out its influence on the pH value, and baseline measurement was performed before the experiment started. Then, to ascertain the glycolytic flow and glycolytic capability of the investigated cells, glucose (10 mM), oligomycin (1 μM), an inhibitor of oxidative phosphorylation, and 2-deoxyglucose (2-DG; 50 mM) (both from Sigma Aldrich, USA) were utilized. Seahorse Biosciences Wave software was used to evaluate the data.

### Lactate Assay

The lysates were centrifuged at 2000×g for 15 min at 4°C after the cells had been collected and dispersed using an ultrasonic disintegrator. In accordance with the manufacturer's recommendations, the supernatant was collected, and the level of lactic acid was determined using a lactic acid detection kit (Nanjing Jiancheng Bioengineering Institute, Nanjing, China).

### 2-NBDG uptake assay

After the indicated KCs treatments, the old medium was replaced with glucose-free medium, and the cells were then deprived of glucose for 2 to 3 h. Following a glucose-free DMEM wash, the cells were then incubated under the aforementioned conditions for an additional 30 min in DMEM with or without 100 μM 2-NBDG (Thermo Fisher Scientific, USA), rinsed in 1× PBS, and immediately examined by flow cytometry.

### ATP analysis

The intracellular ATP concentration was measured using an Enhanced ATP Assay Kit (#S0027, Beyotime Biotechnology, Shanghai, China). Cells were plated in white 6-well tissue culture microplates at 1.5×10^5^ cells/well and treated with the indicated treatments. At the appropriate time, 200 μl/well lysis buffer was added. The lysed cells were centrifuged at 12000 g and the total protein concentration of the supernatant was determined by a BCA Kit (Beyotime Biotechnology, Shanghai, China). The supernatant was then used to measure ATP levels. The luminescence intensity of each well was measured using an EnSpireTM 2300 Multilabel reader (PerkinElmer, Waltham, MA, USA).

### Chromatin immunoprecipitation (ChIP) assay

ChIP assays were performed using a Simple ChIP Plus Sonication Chromatin IP Kit (#56383, Cell Signaling Technology, USA) in accordance with the manufacturer's instructions. Briefly, cells were lysed after formaldehyde fixation. To obtain 200-1000 bp chromatin fragments, the chromatin underwent ultrasonic treatment. Anti-K17 (sc-393002, Santa Cruz, USA) was incubated with the recovered supernatant fraction overnight on a rotor at 4°C. The precipitated protein‒DNA complexes were recovered using Protein G Magnetic Beads at 4°C for 16 h. Afterward, phenol/chloroform was used to clean the DNA. Real-time PCR was then used to quantify the quantities of the particular DNA fragment and standardize them against the genomic DNA preparation from the same cells. The primers for LDHA were designed to span -2000 to +100 bp of the promoter. Details of the primer sequences are listed in Supplementary Table S1. Each group was made in triplicate.

### Luciferase reporter assay

HEK293T cells were seeded in 24-well plates at 60% confluence, transfected with K17 overexpression plasmid using Lipofectamine 3000 and then cotransfected with pRL-TK and the LDHA promoter (pGL3-basic-LDHA and pGL3-basic-LDHA F1/R1^-/-^). After transfection for 12 h, the cells were transfected with pGFP-N1-K17 and treated with BI-D1870. After 48 h, the cells were lysed in Passive Lysis Buffer (Promega, Madison, USA), and the luciferase activity was determined with a luminescence plate reader (PerkinElmer) using a dual-luciferase assay system (Promega, Madison, USA). The results from each experiment were obtained from the average of triplicate experiments.

### Small interence RNA, plasmids and established stable cells

siRNA oligos against human K17 and ENO1 were purchased from GenePharma (Shanghai, China). Sequences of the siRNAs are listed in Supplementary Table S2. K17 and its mutants, K17 S44A and K17 S44D were subcloned into the pEGFP-N1 empty vector. siRNA and plasmid transfection were performed using the Invitrogen Lipofectamine RNAiMAX Kit (Invitrogen, Carlsbad, USA) according to the manufacturer's instructions. HaCaT cells were infected in the presence of polybrene with the lentivirus that was produced and selected with 0.5 μg/mL puromycin to establish K17-overexpressing stable cells.

### Real-time quantitative PCR

Total RNA was extracted from cell or tissue samples using TRIzol Reagent (Invitrogen, Carlsbad, USA) in accordance with the manufacturer's instructions. Using a PrimeScriptTM RT Reagent Kit, reverse transcription of RNA samples into cDNA was performed (Takara, Otsu, Japan). Real‒time quantitative PCR (RT‒qPCR) was conducted using SYBR Green PCR Master Mix (Takara, Otsu, Japan) with a CFX384 real-time quantitative PCR detection system (Bio-Rad, Hercules, CA). Primers are listed in Supplementary Table S3. All reactions were run in triplicate for at least three independent experiments. The relative gene expression levels were normalized to human β-Actin or mouse β-Actin and calculated using the comparative Ct (2^ -∆∆CT^) method.

### Western blot analysis

Cell lysates were prepared using RIPA lysis buffer (Runde Biologicals Ltd, China) supplemented with 1 mM phenyl-methylsulfonyl fluoride and a phosphatase inhibitor. Nuclear and cytoplasmic proteins were obtained by a nuclear and cytoplasmic protein extraction kit (#P0028, Beyotime Biotechnology). The protein concentration was quantified by a Pierce BCA Protein Assay Kit (Pierce Biotechnology, Waltham, USA). Then, 15-20 µg of cell lysate samples were separated by 10-15% SDS‒PAGE, transferred to PVDF membranes (Millipore, Burlington, USA), blocked for 1 h with blocking buffer, and incubated with the following primary antibodies overnight at 4°C: anti-K17 (1:1000, ab53707, Abcam, UK), anti-ENO1 (1:1000, ab227978, Abcam, UK), anti-HK2 (1:1000, ab209847, Abcam, UK), anti-PKM2 (1:1000, #4053, Cell Signaling Technology, USA), anti-PFKM (1:1000, ab154804, Abcam, UK), anti-phospho-K17 (Ser44) (1:1000, #3519, Cell Signaling Technology, USA), anti-PCNA (1:1000, #13110, Cell Signaling Technology, USA), anti-RSK1 p90 (phospho S380) (1:1000, ab32203, Abcam, UK), anti-RSK1 (1:1000, ab126818, Abcam, UK), anti-Lamin A/C (1:1000, #4777, Cell Signaling Technology, USA), and anti-β-Actin (1:5000, CW0096M, CWBIO, China). The membranes were washed and then incubated for 1 h at room temperature with an HRP-conjugated secondary antibody. Using Image Lab software version 5.2.1, protein expression levels were measured (Bio-Rad Laboratories, Inc.).

### Co-immunoprecipitation

Cell pellets were collected and subsequently lysed in a lysis buffer containing Halt Protease and Phosphatase Inhibitor Cocktail (Thermo Fisher Scientific, USA). Cell lysates were then incubated with anti-K17 (sc-393002, Santa Cruz, USA) for 3 h at 4°C, followed by incubation with Protein A/G PLUS-Agarose (sc-2003, Santa Cruz, USA) at 4°C on a rocker platform overnight. The beads were washed 4 times with cold PBS. To perform an immunoblot analysis, the supernatant was aspirated and discarded, and the pellet was resuspended in 1x electrophoresis sample buffer.

### Hematoxylin and eosin (H&E) staining

Tissue samples from humans and mice were fixed, paraffin-embedded, cut into 8 µm slices, and then deparaffinized using EZ-DeWaxTM (BioGenex, Fremont, USA). The sections were then stained for histological investigation with hematoxylin and eosin (H&E). Slides were studied using NDP2 viewer software (HAMAMATSU Photonics) after being scanned using a slide scanner (HAMAMATSU Photonics, Shizuoka, Japan).

### Enolase activity

Enolase activity was measured in accordance with the manufacturer's instructions using an Enolase Activity Assay Kit (MAK178-1KT, Sigma, Germany). In brief, reaction buffer was mixed with the samples of cell lysates and incubated at 25°C. The optical density (OD) value was first measured at a wavelength of 570 nm after 5 to 10 minutes, and then measurements were taken every 2 to 3 minutes until the OD value of the most active sample exceeded the value of the highest standard to produce the final measurement. Enolase activity was calculated using an equation described previously 35.

### Protein stability assay

For the protein stability assay, HaCaT cells were treated with 50 μg/ml cycloheximide (CHX, Cell Signaling Technology, USA) for 0, 2, 4, 6, 8, and 10 h before harvesting.

### Cell proliferation assay

At a density of 4×10^3^ cells per well, HaCaT cells were seeded in triplicate into 96-well plates (Guangzhou Jet Bio-Filtration, Guangzhou, China). According to the manufacturer's instructions, the Cell Counting Kit-8 (CCK-8) assay (KeyGEN BioTECH, Nanjing, China) was used to measure the vitality of the cells. A multipurpose microplate reader was used to measure the OD at 450 nm at the relevant time points (Thermo Fisher Scientific, USA). Utilizing the GraphPad Prism program, cell growth curves were generated (GraphPad Software, La Jolla, USA).

### EdU proliferation assay

HaCaT cells were plated in 96-well plates in quadruplicate at a density of 5×10^3^ cells per well. After incubation for 24 h, the cells were transfected with siRNAs, plasmids, or ENOBlock and BI-D1870 for a 48 h period. The EdU Cell Proliferation Assay Kit was used to detect cell proliferation (RiboBio, Guangzhou, China). Fluorescence microscopy was used to determine the percentage of cells that incorporated EdU (Olympus, Tokyo, Japan). EdU-positive cells were counted for each group by selecting five random fields of vision.

### Immunofluorescence and immunohistochemical staining

Biopsies obtained from the lesional skin of psoriasis patients and the skin of healthy controls or from IMQ-induced mice were fixed with a 12% formaldehyde solution and embedded in paraffin. For immunofluorescence staining, cells or skin biopsy specimens were incubated with the following primary antibodies overnight at 4°C: anti-K17 (1:1000, ab53707, Abcam, UK), anti-ENO1 (1:400, ab227978, Abcam, UK), and anti-Ki67 (1:1000, ab15580, Abcam, UK). After three washes with PBS, the cells were incubated with Cy3- or FITC-conjugated secondary antibodies (1:200, BioLegend, San Diego, USA), and Hoechst 33258 (1:1000, Solarbio Technology, China) was applied to all cells to label the nuclei. Samples were analyzed using a confocal microscope (LSM880, Carl Zeiss, Germany). For immunohistochemical staining, the tissue sections were incubated with 0.3% H_2_O_2_ for 10 min and then incubated with one of the following primary antibodies: anti-K17 (1:1000, ab53707, Abcam, UK) or anti-ENO1 (1:400, ab227978, Abcam, UK) overnight at 4°C. Sections were subsequently incubated with an HRP-labeled goat anti-mouse/rabbit antibody (CWBIO, Peking, China) for 1 h at room temperature. DAB (Gene Tech, Shanghai, China) was used to detect biotinylated antibodies.

### Statistical analyses

Each experiment was performed at least in triplicate. Statistical analyses were performed using GraphPad Prism software version 7.0 (GraphPad, La Jolla, USA). For experiments with more than two groups, the differences between groups were compared using Student's t test or one-way ANOVA as indicated in the figure legends (*p<0.05, **p<0.01, and ***p<0.001). FlowJo v10 was used for flow cytometry data analyses. The number of sampled units, n, is indicated in the figure legends.

## Results

### Elevated K17 expression facilitated glycolysis in psoriatic KCs

**Figure 1:**
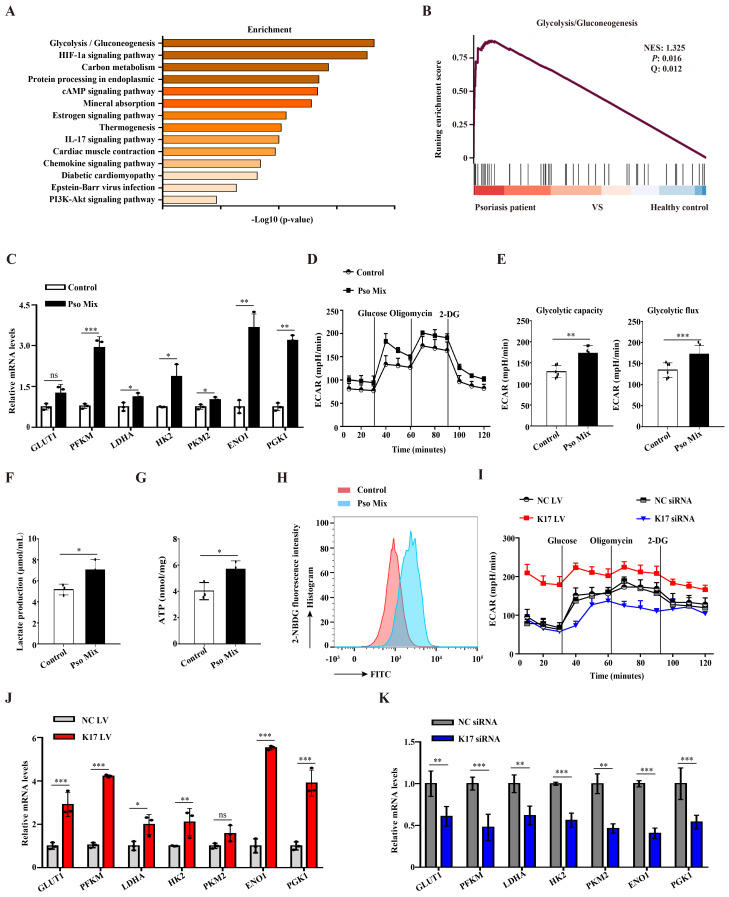
** Elevated K17 expression facilitated glycolysis in psoriatic KCs.** (**A**) Graphs demonstrate the most highly enriched Gene Ontology categories in the single-cell sequencing data from keratinocytes (KCs) of psoriasis patients versus those of healthy controls (*n* = 3). Enriched pathways are listed by their gene count and *p* value. (**B**) Gene set enrichment analysis of single-cell sequencing data from KCs of 3 patients with psoriasis versus those of 5 healthy controls. (**C**) mRNA expression of the key genes associated with glycolysis in Pso Mix-treated KCs (*n* = 3).** (D, E)** The extracellular acid ratio (ECAR), (**F**) extracellular lactate production, (**G**) intracellular ATP levels and (**H**) the uptake of glucose were analyzed in Pso Mix-treated KCs. (*n* = 3 for each group). (**I**) The ECAR of K17-overexpressing KCs and K17 siRNA-transfected KCs was measured using Seahorse XF. 2-DG, 2-deoxyglucose. (**J, K**) mRNA expression of the key genes associated with glycolysis in K17-overexpressing KCs and K17 siRNA-transfected KCs (*n* = 3). Data are presented as the mean ± SEM *(n =* 3-5*)*. *p<0.05, **p<0.01, ***p<0.001, ns, not significant. *p* values were calculated by unpaired Student's *t test*. All experiments were repeated at least three times.

Glycolysis provides a necessary source of energy for proliferating cells, and abnormal glycolysis has been found in numerous diseases. We first evaluated the changes in the expression of genes associated with glycolysis using single-cell RNA-seq (scRNAseq, www.ebi.ac.uk/arrayexpress/experiments/E-MTAB-8142. PMID: 33479125) of skin tissues from 5 healthy individuals and 3 psoriasis patients. In total, 51000 healthy KCs and 49784 psoriatic KCs were extracted and further analyzed. KEGG enrichment and GSEA enrichment data showed that the glycolytic pathway was significantly enriched in psoriatic KCs (**Figure 1A and 1B**). To further explore the results of abnormal glycolysis in psoriatic lesions, we evaluated the glycolysis profile in epidermal KCs from psoriasis patients. RT‒qPCR results showed that KCs from psoriasis patients expressed higher mRNA levels of glycolytic transporters (*GLUT1*) and glycolytic enzymes (*PFKM*, *LDHA*, *HK2*, *PKM2*, *ENO1* and *PGK1*) than those from healthy skin (**Supplementary Figure 1A**). Furthermore, we used a psoriasis-associated cytokine mixture (Pso Mix) (IL-17, TNF-α, IL-1α, OSM and IL-22) to stimulate primary KCs to mimic the psoriatic profile. Glycolytic transporters and glycolytic enzymes were also increased in Pso Mix-treated KCs (**Figure 1C**). The ECAR of these cells was tested by an XF96 Extracellular Flux Analyzer. We found that both the glycolytic flux and glycolytic capacity of Pso Mix-treated KCs were significantly higher than those of controls (**Figure 1D and 1E**). Consistently, lactate production (**Figure 1F**), ATP synthesis (**Figure 1G**) and glucose uptake (**Figure 1H**) were also increased in Pso Mix-treated KCs. These results suggested that glycolysis is enhanced in psoriatic KCs.

Previously, we demonstrated that K17 can regulate KCs proliferation in psoriasis. In this section, we aimed to investigate whether K17 can modulate KCs glycolytic metabolism in psoriasis. First, K17-knockdown and K17-overexpressing KCs were established by using siRNA transfection and lentivirus transfection, respectively. We found that the levels of ECAR increased in K17-overexpressing KCs but inversely decreased in K17-knockdown cells (**Figure 1I and Supplementary Figure 1B**). Consistently, lactate production (**Supplementary Figure 1C**), ATP synthesis (**Supplementary Figure 1D**) and glucose uptake (**Supplementary Figure 1E and 1F**) were increased in K17-overexpressing KCs, while K17 knockdown decreased their levels. Additionally, the mRNA expression of glycolytic transporters and glycolytic enzymes was also increased in K17-overexpressing KCs (**Figure 1J**) but inversely decreased in K17-knockdown KCs (**Figure 1K**). Taken together, these findings suggest that K17 regulates KC glycolysis in psoriasis.

### K17 enhances glycolysis by upregulating the expression and activity of ENO1

**Figure 2:**
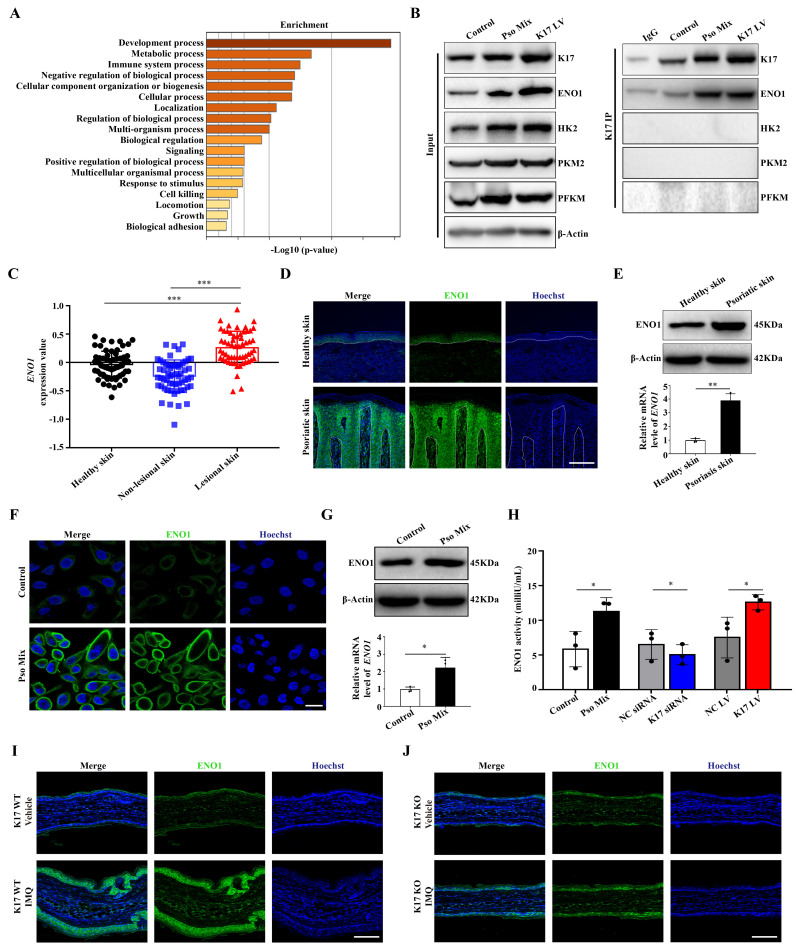
** K17 enhances glycolysis by upregulating the expression and enzyme activity of ENO1.** (**A**) The graphs demonstrate the most highly enriched Gene Ontology categories in the proteomic data of K17-overexpressing keratinocytes (KCs). (**B**) Co-IP assay of K17-binding protein in Pso Mix-treated KCs and K17-overexpressing KCs (*n* = 3 experiments). (**C**) Gene expression profile in the GEO dataset (GDS4602) was analyzed for the mRNA expression of *ENO1* in psoriatic lesional skin *(n =* 57*)*, psoriatic nonlesional skin *(n =* 56*)* and healthy controls *(n =* 63*)*. (**D**) Immunofluorescence staining of ENO1 in the lesional skin of psoriasis patients (*n* = 3) and skin of healthy controls (*n* = 3). ENO1 (green) and Hoechst (blue). Scale bar, 50 μm. (**E**) ENO1 protein and mRNA levels in psoriatic lesional skin (*n* = 3) vs. healthy skin (*n* = 3). (**F**) Immunofluorescence staining of ENO1 in KCs treated with Pso Mix. ENO1 (green) and Hoechst (blue). Scale bar, 10 μm. (**G**) ENO1 protein and mRNA levels in KCs treated with Pso Mix. (**H**) Relative activity of ENO1 in KCs treated with Pso Mix, K17 siRNA-transfected KCs and K17-overexpressing KCs. (**I, J**) Immunofluorescence staining of ENO1 in IMQ-induced psoriasis-like mouse lesional skin of K17 WT and K17 knockout mice (*n* = 5). ENO1 (green) and Hoechst (blue). Scale bar, 50 μm. Data are presented as the mean ± SEM *(n =* 3-5*)*. *p<0.05, **p<0.01, ***p<0.001, ns, not significant. *p* values were calculated by unpaired Student's *t test*. All experiments were repeated at least three times.

To explore the precise molecular mechanisms by which K17 regulates glycolysis, Co-IP combined with mass spectrometry was used in K17-overexpressing KCs. The results from screening the predicted interacting proteins by mass spectrometry and GO enrichment assays showed that the molecular biological processes of proteins interacting with K17 were significantly enriched in metabolic processes (**Figure 2A**). Meanwhile, the mass spectrometry results showed that ENO1, a key enzyme of glycolysis, was involved in glucose metabolism, which was further revealed by Co-IP that ENO1 is a novel candidate interacting protein of K17 (**Figure 2B**). Moreover, the immunofluorescence results showed that K17 and ENO1 were colocalized in KCs treated with Pso Mix and in K17-knockin KCs (**Supplementary Figure 2A**). These results indicate that K17 can directly interact with ENO1 and may contribute to glycolysis.

Next, to investigate whether ENO1 participates in the development of psoriasis, we detected the expression of ENO1 in lesions from psoriasis patients. GEO dataset analysis confirmed that *ENO1* expression is increased in psoriatic lesional skin (*n* = 57) compared to non-lesional skin (*n* = 56) or healthy controls (*n* = 63) (GDS4602) (**Figure 2C**). Immunofluorescence (**Figure 2D**), Western blotting, RT‒qPCR (**Figure 2E**) and IHC staining (**Supplementary Figure 2B**) showed that ENO1 expression was dramatically increased in the epidermis of psoriasis patients compared to normal controls. Consistently, we found that treatment with Pso Mix elevated the protein and mRNA levels of ENO1 in KCs *in vitro* (**Figure 2F and 2G**), which was accompanied by increased ENO1 activity (**Figure 2H**). These results suggested that both the expression and activity of ENO1 were increased in psoriasis.

Then, we investigated whether K17 can modulate ENO1 expression and activity in KCs. The results showed that ENO1 expression was upregulated in K17-overexpressing KCs and downregulated in KCs with K17 knockdown (**Supplementary Figure 2C and 2D**). Moreover, K17 overexpression induced ENO1 activation *in vitro*, while K17 knockdown inhibited ENO1 activation (**Figure 2H**). Furthermore, in a mouse model of imiquimod (IMQ)-induced skin inflammation, the results showed that ENO1 expression was depressed in K17 knockout mice (**Figure 2I and 2J**). Taken together, these data indicate that K17 can directly induce elevated expression and activation of ENO1 in psoriasis.

### Loss of ENO1 inhibits glycolysis and proliferation of psoriatic KCs

**Figure 3:**
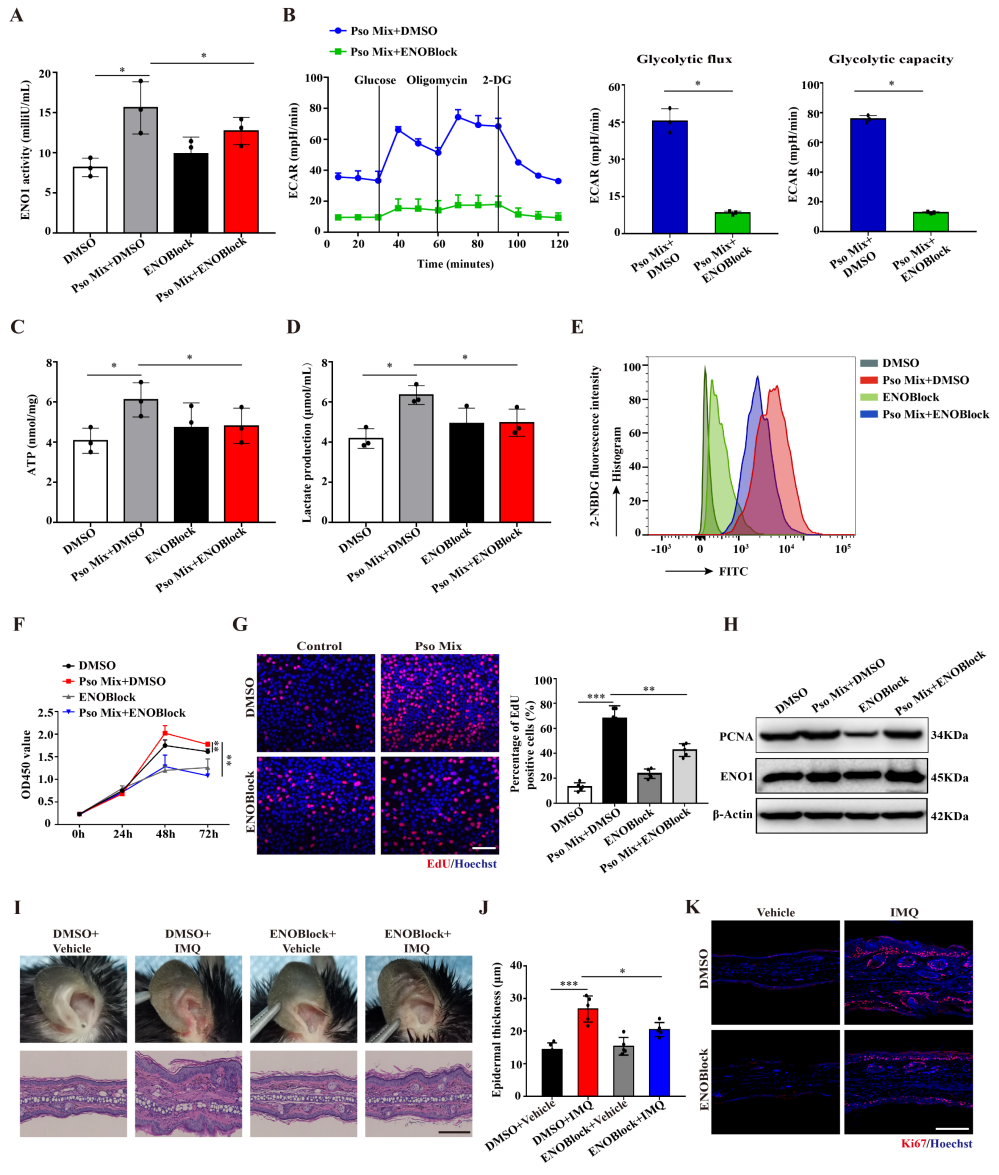
** Loss of ENO1 inhibits glycolysis and proliferation of psoriatic KCs.** (**A**) Relative ENO1 activity in keratinocytes (KCs) treated with the ENO1 inhibitor ENOBlock (*n* = 3). (**B**) The extracellular acid ratio (ECAR), (**C**) intracellular ATP levels, (**D**) extracellular lactate production and (**E**) the uptake of glucose were analyzed in KCs treated with the ENO1 inhibitor ENOBlock. (*n* = 3). (**F**) Cell proliferation was analyzed by Cell Counting Kit-8 (CCK8) assay, (**G**) EdU assay, the percentage of EdU positive cells and (**H**) PCNA protein levels in KCs treated with the ENO1 inhibitor ENOBlock. (**I**) Ear phenotype and H&E staining of lesional skin sections. Scale bar, 10 μm. (**J**) Epidermal thickness and (**K**) immunofluorescence staining of Ki67 in lesional skin from ENO1 inhibitor ENOBlock-treated IMQ-induced psoriasis-like mice; DMSO was used as a control (*n* = 3-5). Ki67 (red) and Hoechst (blue). Scale bar, 50 μm. Data are presented as the mean ± SEM *(n =* 3-5*)*. *p<0.05, **p<0.01, ***p<0.001. *p* values were calculated by unpaired Student's *t test.* All experiments were repeated at least three times.

To explore the effect of ENO1 on KCs dysfunction in psoriasis, we established KC models with ENO1 silencing using siRNA or inhibition by an ENO1 inhibitor (ENOBlock, a nonenzymatic active site inhibitor of ENO1) (**Supplementary Figure 3A and Figure 3A**). Then, we performed the ECAR assay with an XF96 Extracellular Flux Analyzer to detect glycolytic flux. As shown in **Figure 3B** and **Supplementary Figure S3B**, the levels of ECAR were decreased when ENO1 was inhibited or silenced. Consistently, ENO1 inhibition and silencing also decreased ATP synthesis, lactate acid production and glucose consumption (**Figure 3C-E and Supplementary Figure 3C-E**). These results suggested that ENO1 regulates glycolytic flux in psoriatic KCs.

Next, we investigated whether ENO1 affects the proliferation of KCs. CCK8 and EdU assays showed that ENO1 inhibition and silencing suppressed the proliferation of KCs induced by Pso Mix (**Figure 3F and 3G and Supplementary Figure 3F and 3G**). Meanwhile, the levels of PCNA, a proliferation marker, were also decreased in KCs with ENO1 inhibition or silencing (**Figure 3H and Supplementary Figure 3H**). Finally, by establishing a mouse model of IMQ-induced psoriasis, we found that local application of ENOBlock (**Figure 3I and 3J**) or ENO1 siRNA (**Supplementary Figure 4A and 4B**) relieved the symptoms of erythema and scaling, reduced the thickness of the epidermis (**Supplementary Figure 4C and 4D**), downregulated Ki67 expression (**Figure 3K and Supplementary Figure 4E-G**) and inhibited mRNA expression of proliferation-related genes such as *K17*, *K16*, *PCNA* and* Cyclin D1* (**Supplementary Figure 4H and 4I**). Altogether, our results suggest that ENO1 directly regulates glycolysis and the proliferation of KCs, thus exacerbating the development of psoriasis.

### ENO1 maintains the phosphorylation of K17-Ser^44^, which regulates glycolysis and proliferation of KCs in psoriasis

Given that ENO1 can directly bind to K17, in this section, we aimed to investigate whether ENO1 can modulate K17. First, we tested the stability of K17 in KCs upon treatment with the protein synthesis inhibitor cycloheximide (CHX) in control, Pso Mix-treated, and ENO1 siRNA-treated HaCaT cells. As shown in **Supplementary Figure 5A**, the K17 protein level was significantly reduced in control cells, and treatment with Pso Mix partially rescued the CHX-induced reduction in K17, while ENO1 silencing did not yield similar trends to those observed in the control group. These findings indicated that ENO1 is not involved in the maintenance of the stability of K17.

**Figure 4:**
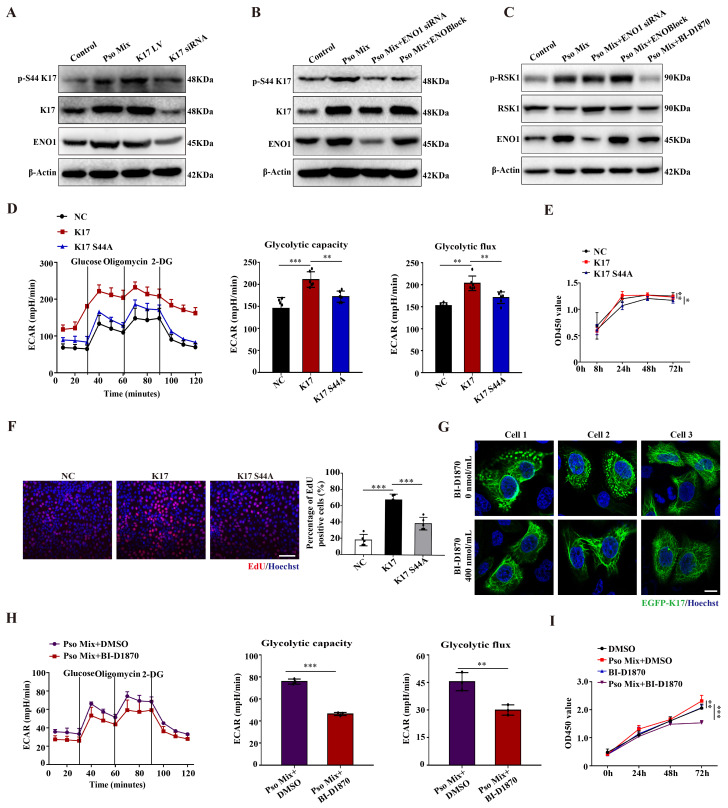
** ENO1 promotes cell glycolysis and proliferation by regulating the phosphorylation of K17.** (**A**) Protein expression of phosphorylated K17, K17 and ENO1 in keratinocytes (KCs) treated with Pso Mix, K17-overexpressing KCs and K17 siRNA-transfected KCs. (**B**) Phosphorylation of K17 in KCs treated with Pso Mix and ENO1 siRNA-transfected or ENOBlock-treated KCs. (**C**) RSK1 kinase activity in Pso Mix and ENO1 siRNA-transfected, ENOBlock-treated and BI-D1870-treated KCs. (**D**) The extracellular acid ratio (ECAR) of K17 S44A mutant-transfected KCs. (**E**) Cell proliferation was analyzed using the CCK8 assay, (**F**) EdU assay and the percentage of EdU positive cells in mutant K17 S44A-transfected KCs. (**G**) Immunofluorescence staining for the punctate form of EGFP-K17 in BI-D1870-treated EGFP-K17-transfected KCs (*n* = 3). K17 (green) and Hoechst (blue). Scale bar, 10 μm. (**H**) The ECAR of Pso Mix and BI-D1870-treated KCs. (**I**) Cell proliferation was analyzed by a CCK8 assay in BI-D1870-treated KCs. Data are presented as the mean ± SEM *(n =* 3-5*)*. *p<0.05, **p<0.01, ***p<0.001. *p* values were calculated by unpaired Student's *t test*. All experiments were repeated at least three times.

Xiaoou Pan et al reported that p90 ribosomal protein S6 kinase 1 (RSK1) can phosphorylate K17 at amino acid position 44 36. Our results showed that K17-Ser^44^ phosphorylation was significantly elevated in psoriasis patients compared to normal skin (**Supplementary Figure 5B**). Meanwhile, Pso Mix induced the phosphorylation of K17-Ser^44^, and the phosphorylation level was significantly affected by the level of K17 expression (**Figure 4A**). Furthermore, we determined whether the phosphorylation of K17-Ser^44^ was affected by ENO1. The results showed that the phosphorylation of K17-Ser^44^ was significantly repressed in KCs treated with ENO1 siRNA or ENOBlock (**Figure 4B**). However, the expression of phosphorylated RSK1 scarcely changed in the ENOBlock or ENO1 siRNA group compared to the Pso Mix group (**Figure 4C and Supplementary Figure 5C**), suggesting that ENO1 can regulate the phosphorylation of K17-Ser^44^ with independence of RSK kinase.

To further investigate the effect of K17-Ser^44^ phosphorylation on the function of KCs, we constructed mutant K17 overexpression plasmids pEGFP-N1-K17 S44A and pEGFP-N1-K17 S44D at amino acid position 44 (**Supplementary Figure 5D**), and the results showed that K17 S44A significantly repressed the phosphorylation of K17, which manifested as punctate form changes caused by phosphorylation (**Supplementary Figure 5E and 5F**). Furthermore, we found that mutant K17 S44A repressed the ECAR level (**Figure 4D**), indicating that phosphorylation of K17-Ser^44^ promotes glycolysis in KCs. Additionally, CCK8 and EdU assay results showed that the proliferation of KCs was markedly repressed by the mutant K17 S44A (**Figure 4E and 4F**). To investigate further, we established KCs by blocking the phosphorylation of K17-Ser^44^ using the RSK1 kinase inhibitor BI-D1870 (400 nmol/mL) (**Figure 4G and Supplementary Figure 5G and 5H**). Consistently, the BI-D1870 inhibitor repressed the ECAR level (**Figure 4H**), accompanied by decreased proliferation of KCs (**Figure 4I and Supplementary Figure 5I**). Collectively, these data document that ENO1 modulates the phosphorylation of K17, which further contributes to the glycolysis and proliferation of KCs in psoriasis.

### ENO1 maintains the phosphorylation of K17-Ser^44^ to act as a transcription factor

**Figure 5:**
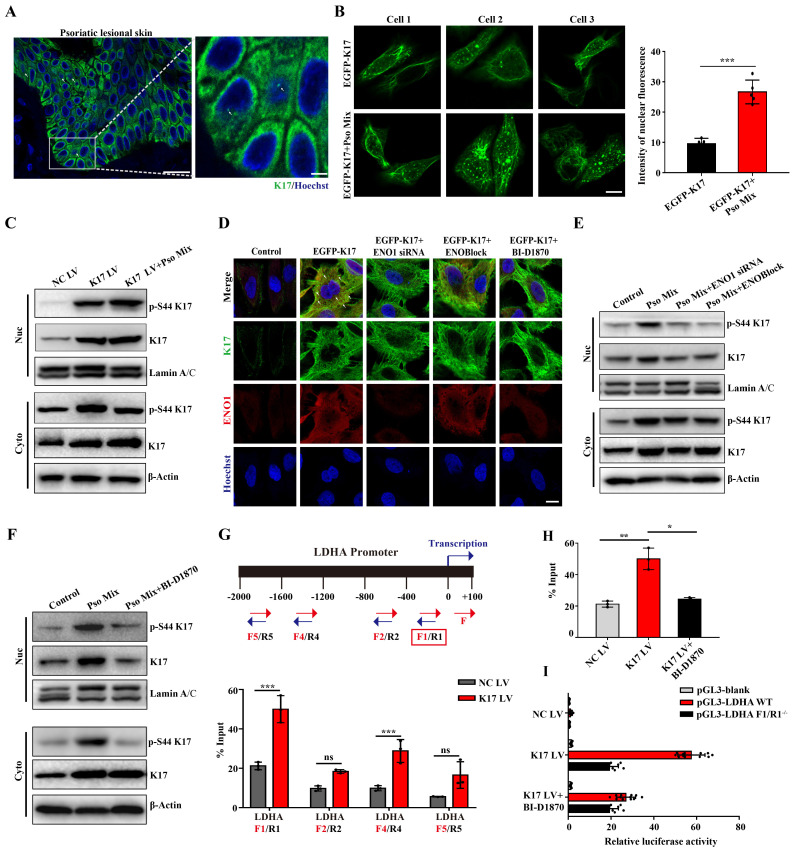
** Phosphorylation of K17-Ser^44^ promotes its translocation to the nucleus.** (**A**) Immunofluorescence staining for K17 in the nucleus of keratinocytes (KCs) of lesional skin from psoriasis patients (*n* = 3). K17 (green) and Hoechst (blue). Scale bar, 10 μm. (**B**) Immunofluorescence staining of K17 distributed in the nuclei of KCs transfected with EGFP-K17 and treated with Pso Mix and the intensity of nuclear fluorescence per unit area. Scale bar, 10 μm. (**C**) K17 and phosphorylated K17-Ser^44^ protein levels in the nucleus and cytoplasm of K17-overexpressing and Pso Mix-treated KCs. (**D**) Immunofluorescence staining for the punctate form of EGFP-K17 in ENO1 siRNA-transfected, ENOBlock- or BI-D1870-treated EGFP-K17-transfected KCs (*n* = 3). K17 (green), ENO1 (red) and Hoechst (blue). White arrows: the punctate and diffuse forms of EGFP-K17. Scale bar, 10 μm. (**E**) K17 and phosphorylated K17-Ser^44^ protein levels in the nucleus and cytoplasm of Pso Mix and ENO1 siRNA-transfected or ENOBlock-treated KCs. (**F**) K17 and phosphorylation of K17-Ser^44^ protein levels in the nucleus and cytoplasm of Pso Mix and RSK kinase inhibitor BI-D1870-treated KCs. Data are presented as the mean ± SEM *(n = 3-5)*. ChIP assays of K17 binding to the *LDHA* promoter in (**G**) K17-overexpressing KCs and (**H**) BI-D1870-treated K17-overexpressing KCs. (**I**) Luciferase reporter assays in HEK293T cells to test the *LDHA* WT and mutants. *p<0.05, **p<0.01, ***p<0.001, ns, not significant. *p* values were calculated by unpaired Student's *t test*. All experiments were repeated at least three times.

Emerging evidence suggests that K17 is not exclusively present in the cytoplasm but also localizes to the nucleus. Consistent with these findings, K17 was overexpressed, and a subset of the overall K17 population was distributed in the nucleus of epidermal KCs in the lesional skin of psoriasis patients (**Figure 5A**), which was different from the reticular forms of the cytoskeleton in the cytoplasm in normal skin. To investigate further, we constructed the K17-overexpressing plasmid pEGFP-N1-K17 and transiently transfected KCs with liposome 3000. Notably, a portion of the EGFP-K17 fusion protein exhibited diffuse or punctate patterns in the nucleus following transfection of KCs with the EGFP-K17-overexpressing vector for 24 h. Pso Mix cytokines further increased the levels of the diffuse or punctate pattern of intranuclear K17 (**Figure 5B**). These results were further confirmed by an immunoblotting technique with cytoplasmic and nuclear separation (**Figure 5C**). These results indicate that phosphorylated K17 is prone to translocate into the nucleus, which is specifically found in psoriatic KCs.

Furthermore, considering that the phosphorylation of K17-Ser^44^ can be regulated by both RSK1 kinase and ENO1, we aimed to investigate the role of these two kinases in K17 translocation into the nucleus. We found that when the phosphorylation of K17-Ser^44^ was blocked with ENO1 siRNA, ENOBlock or BI-D1870, the punctate pattern of K17 was dramatically eliminated in KCs with the K17 overexpression plasmid pEGFP-N1-K17 (**Figure 5D**), and the level of phosphorylation of K17-Ser^44^ was also decreased in both the nucleus and cytoplasm of KCs (**Figure 5E and 5F**). Collectively, these results suggest that ENO1 regulates the nuclear translocation of K17 by maintaining its phosphorylation.

Finally, to study whether phosphorylated K17 functions as a transcription factor, we performed a ChIP assay to detect the binding activity of K17 to the promoter regions of LDHA, the key enzyme downstream of ENO1 in the glycolytic pathway. The ChIP results revealed that K17 can directly interact with *LDHA* gene promoters (**Figure 5G and 5H and Supplementary Figure 6**). To further confirm this observation, we generated *LDHA* WT and mutant promoter reporters LDHA F1/R^-/-^. K17 increased the reporter activity of LDHA, while mutation of LDHA largely abolished K17-induced promoter reporter activity (**Figure 5I**). Collectively, these results suggested that ENO1 maintains the phosphorylation of K17-Ser^44^ to promote its nuclear translocation, which further regulates the transcription of LDHA, a key enzyme of glycolysis, and thereby fosters glycolysis and KCs proliferation.

### Inhibiting the phosphorylation of K17-Ser^44^ significantly alleviated skin inflammation in IMQ-induced psoriasis-like mice

**Figure 6:**
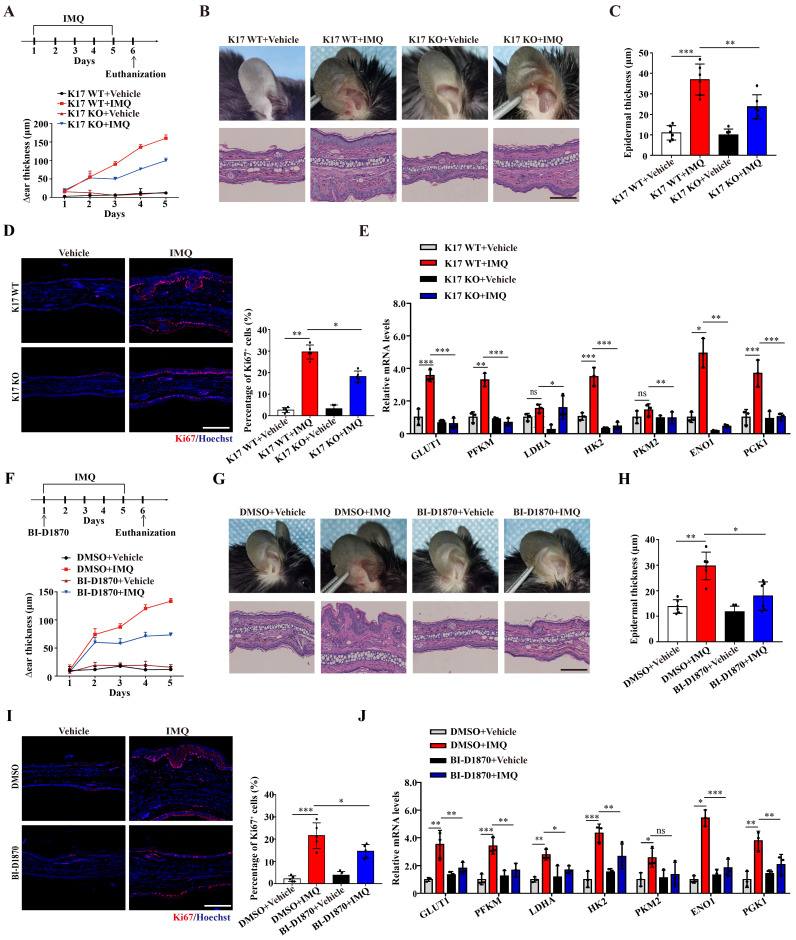
** Inhibiting the phosphorylation of K17 significantly alleviated skin inflammation in IMQ-induced psoriasis-like mice.** (**A**) Schematic of the animal experimental protocol for IMQ-induced psoriasis-like inflammation in K17 KO and K17 WT mice and examination of Δear thickness. (**B**) Psoriatic phenotype and hematoxylin and eosin (H&E) staining, Scale bar, 10 μm. (**C**) Epidermal thickness and (**D**) immunofluorescence staining of Ki67 and the percentage of Ki67^+^ cells. Ki67 (red) and Hoechst (blue). Scale bar, 50 μm. (**E**) Relative mRNA expression of* GLUT1*, *PFKM*,* LDHA*, *HK2*, *PKM2*, *ENO1* and *PGK1* in IMQ-induced psoriasis-like inflammation in K17 KO and K17 WT mice. (**F**) Schematic of the animal experimental protocol in mice with depression of K17 phosphorylation and examination of Δear thickness, *n* = 3 per group. (**G**) Ear phenotype and H&E staining. Scale bar, 10 μm. (**H**) Epidermal thickness and (**I**) immunofluorescence staining of Ki67 and the percentage of Ki67^+^ cells. Ki67 (red) and Hoechst (blue). Scale bar, 50 μm. (**J**) Relative mRNA expression of* GLUT1*, *PFKM*,* LDHA*, *HK2*, *PKM2*, *ENO1* and *PGK1*. The study involved 3-5 mice per group. Data are presented as the mean ± SEM *(n =* 3-5*)*. *p<0.05, **p<0.01, ***p<0.001, ns, not significant. *p* values were calculated by unpaired Student's *t tes*t or one-way ANOVA. All experiments were repeated at least three times.

We next examined the role of K17-Ser^44^ phosphorylation in the pathogenesis of psoriasis in an IMQ-induced psoriasis model. By establishing an IMQ-induced psoriasis-like model using K17 knockout mice, we found that Δear thickness, swelling and scaling were alleviated in K17 KO mice, and the relieved psoriasis phenotype was confirmed at the cellular level by H&E staining of KCs from K17 KO mice (**Figure 6A-C**). Additionally, the immunofluorescence results showed that Ki67 expression was decreased in K17 KO mice in the IMQ-induced psoriasis-like mouse model (**Figure 6D**). Consistently, RT‒qPCR results showed that K17 KO also downregulated the mRNA levels of the proliferation markers *K17, K16, PCNA, Cyclin D1 (***Supplementary Figure 7A)** and key enzymes (*GLUT1*, *PFKM*,* LDHA*, *HK2*, *PKM2*, *ENO1* and *PGK1)* in the IMQ-induced psoriasis-like model (**Figure 6E**). These results confirm that K17 deficiency suppresses glycolysis and proliferation of KCs and alleviates psoriasis *in vivo*.

Next, we blocked the phosphorylation of K17 in an IMQ-induced psoriasis-like model using local application of an RSK kinase inhibitor (BI-D1870). Consistent with the K17 KO mice, local application of BI-D1870 relieved the psoriasis phenotype (**Figure 6F-H**). Moreover, Ki67 expression (**Figure 6I**) and the mRNA levels of proliferation markers *K17, K16, PCNA, Cyclin D1 (***Supplementary Figure 7B)** and key enzymes (*GLUT1*, *PFKM*,* LDHA*, *HK2*, *PKM2*, *ENO1* and *PGK1)* were also decreased in epidermal KCs of the IMQ-induced psoriasis-like model with local application of BI-D1870 (**Figure 6J**). Taken together, the results confirmed that phosphorylation of K17 modulates the glycolytic flux and proliferation of KCs and contributes to the pathogenesis of psoriasis.

## Discussion

**Figure 7:**
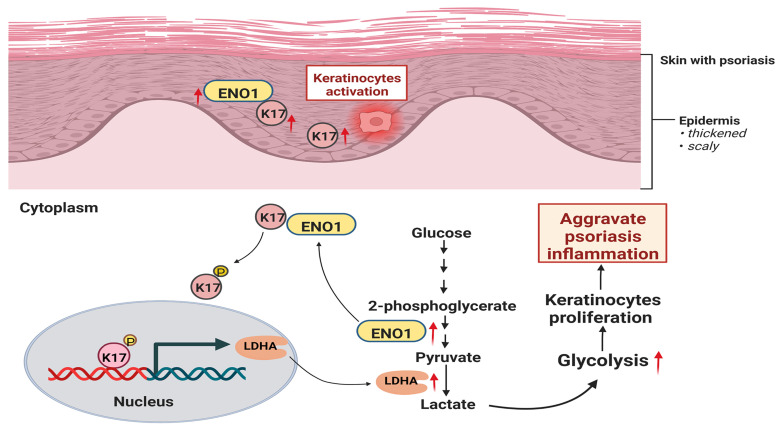
** Graphical summary of K17 in dysregulated proliferation in psoriasis.** In psoriatic lesional skin, keratinocytes (KCs) are activated, and K17 is excessively elevated and interacts with ENO1, which maintains the phosphorylation of K17-Ser^44^ to promote K17 nuclear translocation and further regulates the transcription of the key glycolysis enzyme lactic dehydrogenase A (*LDHA*) to contribute to glycolysis and the proliferation of KCs, thereby exacerbating psoriatic inflammation (Figure was created with Biorender.com).

In the present study, we demonstrate that glycolysis is enhanced in psoriatic KCs and can be regulated by K17. Furthermore, we found that K17 can directly regulate the activation and expression of ENO1, the key enzyme in the glycolysis pathway. Meanwhile, the excessive expression of ENO1 maintains the phosphorylation of K17-Ser^44^, and phosphorylated K17 functions as a transcription factor to promote the glycolysis and proliferation of KCs in psoriasis (**Figure 7**). Taken together, our results indicate that the interaction of K17 and ENO1 boosts the glycolysis and proliferation of KCs and accelerates the process of psoriasis development.

KCs hyperproliferation is a key phenotype associated with the pathogenesis of psoriasis 37, 38. Previous studies on the mechanism of KCs proliferation have mainly focused on cytokines and inflammatory pathways 39, 40. In recent years, many studies have shown that glycolysis is essential for regulating cell proliferation 41, 42. KCs exhibit high levels of glycolysis metabolism and lactate production in psoriasis 43, 44. Yunzi Liu et al found that glycolysis in which the final step of the pathway is mediated by the pyruvate kinase M2 isoform is important for KCs proliferation in psoriasis 45. Here, we found that ENO1, another key enzyme in the glycolysis pathway, was significantly overexpressed and activated in psoriatic KCs. *In vitro* experiments and animal models confirmed that ENO1 was involved in regulating glycolysis and cell proliferation, thus contributing to the development of psoriasis.

Recently, ENO1 was found to play important roles in regulating cell proliferation 46, 47, hypoxia induction and autoimmunity, in addition to its classical role as an enzyme in glycolysis 48, 49. Some studies have suggested that ENO1 can directly activate the PI3K/AKT signaling pathway to promote the proliferation of cancer cells 50, including pancreatic cancer and gastric cancer cells. ENO1 can also regulate the cell cycle and apoptosis of bladder cancer by β-catenin signaling 51. In our study, we found that ENO1 directly regulates KCs proliferation in psoriasis by interacting with K17. Furthermore, our *in vitro* experiments indicated that ENO1 could promote and maintain K17 phosphorylation and translocation into the nucleus. Finally, using an IMQ-induced psoriasis-like mouse model, we confirmed that inhibiting either the expression or activity of ENO1 alleviated the severity of psoriasis.

In the pathogenesis of psoriasis, KCs are always the first responders to environmental stimulation such as trauma and stress. Previous studies suggested that environmental stimulation induces KCs to produce some proinflammatory cytokines and antimicrobial peptides, thus initiating the immune response. In this early stage of psoriatic pathogenesis, K17 is abnormally expressed by environmental stimulation 52 and further activates inflammatory pathways, including STAT3, Erk and NF-κB 53, 54, to induce proinflammatory cytokine production. Thus, these results indicate that K17 induces the expression and activity of ENO1 and may be an early event in psoriasis. In addition, the current study indicates that excessive proliferation of KCs is mainly caused by proinflammatory cytokines, and this may be a downstream biological event in psoriasis 55, 56. Our and previous studies suggested that ENO1 is involved in the proliferation of KCs 57, 58. It is unknown whether ENO1 contributes to the production of proinflammatory cytokines and antimicrobial peptides in KCs. In this study, we found that K17 could promote glycolysis in KCs in psoriasis. On the one hand, K17 directly interacts with ENO1 and induces its activation. On the other hand, ENO1 regulates and maintains the phosphorylation of K17, and phosphorylated K17 acts as a transcription factor and further regulates the transcription of LDHA, a downstream enzyme of ENO1 in the glycolytic pathway. Thus, these results indicate that ENO1-regulated phosphorylation of K17-Ser44 mainly contributes to the progression and exacerbation of psoriasis.

Nevertheless, there still exist some limitations in this study. BI-D1870 is a specific inhibitor of the kinase RSK1, which can phosphorylate K17 on residue serine 44 36 in a growth- and stress-dependent fashion. In a previous study, Byung Min Chung et al used BI-D1870 treatment to investigate the role of K17 phosphorylation in skin tumor cell growth and invasion 59. To date, no other kinase has been found to regulate K17 phosphorylation. Thus, in this study, we used the RSK1 inhibitor BI-D1870 to investigate the role of K17 phosphorylation in psoriasis. Furthermore, to provide stronger evidence from a different angle, we constructed mutant K17 overexpression plasmids pEGFP-N1-K17 S44A and pEGFP-N1-K17 S44D at amino acid position 44, and the results showed that K17 S44A significantly repressed the phosphorylation of K17 and the punctate form changes caused by phosphorylation. In addition, to investigate whether ENO1 induces K17 phosphorylation by regulating RSK1, we examined the role of ENO1 in the expression and activation of RSK1. The results showed that ENO1 inhibition had no effect on the expression and activation of RSK1. Thus, we conclude that ENO1 can directly regulate K17 phosphorylation with independence of RSK1 in psoriasis.

## Conclusion

In summary, our study revealed the mechanism by which K17 interacts with ENO1 to promote glycolysis and proliferation of KCs, mediating the occurrence and development of psoriasis. K17 binds to ENO1, initiates its activation and increases its expression. Conversely, ENO1 regulates and maintains the phosphorylation of K17, which further acts as a transcription factor to promote the transcription of LDHA, amplifying the glycolysis process and leading to hyperproliferation of KCs in psoriasis. This study provides a new perspective for the study of the correlation between proliferation and metabolism.

## Supplementary Material

Supplementary figures and tables.

## Abbreviations

CCK-8: Cell Counting Kit-8
ChIP: Chromatin immunoprecipitation
CHX: Cycloheximide
Co-IP: Co-immunoprecipitation
DMEM: Dulbecco's Modified Eagle's medium
ECAR: Extracellular acidification rate
EDGS: EpiLife Defined Growth Supplement
ENO1: alpha-enolase
ENOBlock: ENO1 inhibitor/blocker
EGFP: Enhanced green fluorescent protein
FBS: Fetal bovine serum
H&E: Hematoxylin and eosin
KCs: Keratinocytes
K17: Keratin 17
LDHA: Lactic dehydrogenase A
OSM: Oncostatin M
PBS: phosphate-buffered saline
RT-qPCR: Real‒time quantitative PCR
RSK1: p90 ribosomal protein S6 kinase 1
scRNAseq: single-cell RNA sequencing data
siRNA: small interfering RNA
2-DG: 2-deoxyglucose

## References

[1] Boehncke WH, Schon MP (2015). Psoriasis. Lancet.

[2] Greb JE, Goldminz AM, Elder JT, Lebwohl MG, Gladman DD, Wu JJ (2016). Psoriasis. Nat Rev Dis Primers.

[3] Hawkes JE, Chan TC, Krueger JG (2017). Psoriasis pathogenesis and the development of novel targeted immune therapies. J Allergy Clin Immunol.

[4] Yu J, Zhao Q, Wang X, Zhou H, Hu J, Gu L (2022). Pathogenesis, multiomics research, and clinical treatment of psoriasis. J Autoimmun.

[5] Fu M, Wang G (2012). Keratin 17 as a therapeutic target for the treatment of psoriasis. J Dermatol Sci.

[6] Zhang X, Yin M, Zhang LJ (2019). Keratin 6, 16 and 17-Critical Barrier Alarmin Molecules in Skin Wounds and Psoriasis. Cells.

[7] Lin Y, Zhang W, Li B, Wang G (2022). Keratin 17 in psoriasis: Current understanding and future perspectives. Semin Cell Dev Biol.

[8] Zhang W, Dang E, Shi X, Jin L, Feng Z, Hu L (2012). The pro-inflammatory cytokine IL-22 upregulates keratin 17 expression in keratinocytes via STAT3 and ERK1/2. PLoS One.

[9] Yang L, Zhang S, Wang G (2019). Keratin 17 in disease pathogenesis: from cancer to dermatoses. J Pathol.

[10] Jin L, Wang G (2014). Keratin 17: a critical player in the pathogenesis of psoriasis. Med Res Rev.

[11] Yang L, Fan X, Cui T, Dang E, Wang G (2017). Nrf2 Promotes Keratinocyte Proliferation in Psoriasis through Up-Regulation of Keratin 6, Keratin 16, and Keratin 17. J Invest Dermatol.

[12] Zhuang Y, Han C, Li B, Jin L, Dang E, Fang H (2018). NB-UVB irradiation downregulates keratin-17 expression in keratinocytes by inhibiting the ERK1/2 and STAT3 signaling pathways. Arch Dermatol Res.

[13] Hobbs RP, DePianto DJ, Jacob JT, Han MC, Chung BM, Batazzi AS (2015). Keratin-dependent regulation of Aire and gene expression in skin tumor keratinocytes. Nat Genet.

[14] Hobbs RP, Batazzi AS, Han MC, Coulombe PA (2016). Loss of Keratin 17 induces tissue-specific cytokine polarization and cellular differentiation in HPV16-driven cervical tumorigenesis *in vivo*. Oncogene.

[15] Kinoshita J, Kawamori R (2002). Gluconeogenesis and glycolysis. Nihon Rinsho.

[16] Kornberg MD, Bhargava P, Kim PM, Putluri V, Snowman AM, Putluri N (2018). Dimethyl fumarate targets GAPDH and aerobic glycolysis to modulate immunity. Science.

[17] Ganapathy-Kanniappan S, Geschwind JF (2013). Tumor glycolysis as a target for cancer therapy: progress and prospects. Mol Cancer.

[18] Chandel NS (2021). Glycolysis. Cold Spring Harb Perspect Biol.

[19] Sreedhar A, Petruska P, Miriyala S, Panchatcharam M, Zhao Y (2017). UCP2 overexpression enhanced glycolysis via activation of PFKFB2 during skin cell transformation. Oncotarget.

[20] Hosseini M, Dousset L, Mahfouf W, Serrano-Sanchez M, Redonnet-Vernhet I, Mesli S (2018). Energy Metabolism Rewiring Precedes UVB-Induced Primary Skin Tumor Formation. Cell Rep.

[21] Pavel P, Leman G, Hermann M, Ploner C, Eichmann TO, Minzaghi D (2021). Peroxisomal Fatty Acid Oxidation and Glycolysis Are Triggered in Mouse Models of Lesional Atopic Dermatitis. JID Innov.

[22] Zezina E, Sercan-Alp O, Herrmann M, Biesemann N (2020). Glucose transporter 1 in rheumatoid arthritis and autoimmunity. Wiley Interdiscip Rev Syst Biol Med.

[23] Lin HC, Chen YJ, Wei YH, Lin HA, Chen CC, Liu TF (2021). Lactic Acid Fermentation Is Required for NLRP3 Inflammasome Activation. Front Immunol.

[24] Blunder S, Pavel P, Minzaghi D, Dubrac S (2021). PPARdelta in Affected Atopic Dermatitis and Psoriasis: A Possible Role in Metabolic Reprograming. Int J Mol Sci.

[25] Tsai ST, Chien IH, Shen WH, Kuo YZ, Jin YT, Wong TY (2010). ENO1, a potential prognostic head and neck cancer marker, promotes transformation partly via chemokine CCL20 induction. Eur J Cancer.

[26] Xu CM, Luo YL, Li S, Li ZX, Jiang L, Zhang GX (2019). Multifunctional neuron-specific enolase: its role in lung diseases. Biosci Rep.

[27] Jiang K, Dong C, Yin Z, Li R, Mao J, Wang C (2020). Exosome-derived ENO1 regulates integrin alpha6beta4 expression and promotes hepatocellular carcinoma growth and metastasis. Cell Death Dis.

[28] Cheng Z, Shao X, Xu M, Zhou C, Wang J (2019). ENO1 Acts as a Prognostic Biomarker Candidate and Promotes Tumor Growth and Migration Ability Through the Regulation of Rab1A in Colorectal Cancer. Cancer Manag Res.

[29] Li HJ, Ke FY, Lin CC, Lu MY, Kuo YH, Wang YP (2021). ENO1 Promotes Lung Cancer Metastasis via HGFR and WNT Signaling-Driven Epithelial-to-Mesenchymal Transition. Cancer Res.

[30] Huang CK, Sun Y, Lv L, Ping Y (2022). ENO1 and Cancer. Mol Ther Oncolytics.

[31] Zhang Y, Li M, Liu Y, Han N, Zhang K, Xiao T (2010). ENO1 protein levels in the tumor tissues and circulating plasma samples of non-small cell lung cancer patients. Zhongguo Fei Ai Za Zhi.

[32] Song Y, Luo Q, Long H, Hu Z, Que T, Zhang X (2014). Alpha-enolase as a potential cancer prognostic marker promotes cell growth, migration, and invasion in glioma. Mol Cancer.

[33] Cao T, Zhang H, Zhou L, Wang Y, Du G, Yao H (2017). *In vitro* cell culture system optimization of keratinocytes from oral lichen planus (OLP) patients. Oral Dis.

[34] Plitzko B, Loesgen S (2018). Measurement of Oxygen Consumption Rate (OCR) and Extracellular Acidification Rate (ECAR) in Culture Cells for Assessment of the Energy Metabolism. Bio Protoc.

[35] Fukano K, Kimura K (2014). Measurement of enolase activity in cell lysates. Methods Enzymol.

[36] Pan X, Kane LA, Van Eyk JE, Coulombe PA (2011). Type I keratin 17 protein is phosphorylated on serine 44 by p90 ribosomal protein S6 kinase 1 (RSK1) in a growth- and stress-dependent fashion. J Biol Chem.

[37] Gran F, Kerstan A, Serfling E, Goebeler M, Muhammad K (2020). Current Developments in the Immunology of Psoriasis. Yale J Biol Med.

[38] Thatikonda S, Pooladanda V, Sigalapalli DK, Godugu C (2020). Piperlongumine regulates epigenetic modulation and alleviates psoriasis-like skin inflammation via inhibition of hyperproliferation and inflammation. Cell Death Dis.

[39] Deng Y, Chang C, Lu Q (2016). The Inflammatory Response in Psoriasis: a Comprehensive Review. Clin Rev Allergy Immunol.

[40] Gao J, Chen F, Fang H, Mi J, Qi Q, Yang M (2020). Daphnetin inhibits proliferation and inflammatory response in human HaCaT keratinocytes and ameliorates imiquimod-induced psoriasis-like skin lesion in mice. Biol Res.

[41] DeBerardinis RJ, Lum JJ, Hatzivassiliou G, Thompson CB (2008). The biology of cancer: metabolic reprogramming fuels cell growth and proliferation. Cell Metab.

[42] Lunt SY, Vander Heiden MG (2011). Aerobic glycolysis: meeting the metabolic requirements of cell proliferation. Annu Rev Cell Dev Biol.

[43] Cibrian D, de la Fuente H, Sanchez-Madrid F (2020). Metabolic Pathways That Control Skin Homeostasis and Inflammation. Trends Mol Med.

[44] Tang W, Long T, Li F, Peng C, Zhao S, Chen X (2021). HIF-1alpha may promote glycolysis in psoriasis vulgaris via upregulation of CD147 and GLUT1. Zhong Nan Da Xue Xue Bao Yi Xue Ban.

[45] Liu YZ, Xu MY, Dai XY, Yan L, Li L, Zhu RZ (2021). Pyruvate Kinase M2 Mediates Glycolysis Contributes to Psoriasis by Promoting Keratinocyte Proliferation. Front Pharmacol.

[46] Diaz-Ramos A, Roig-Borrellas A, Garcia-Melero A, Lopez-Alemany R (2012). alpha-Enolase, a multifunctional protein: its role on pathophysiological situations. J Biomed Biotechnol.

[47] Ji H, Wang J, Guo J, Li Y, Lian S, Guo W (2016). Progress in the biological function of alpha-enolase. Anim Nutr.

[48] Gou Y, Li F, Huo X, Hao C, Yang X, Pei Y (2021). ENO1 monoclonal antibody inhibits invasion, proliferation and clone formation of cervical cancer cells. Am J Cancer Res.

[49] Zhang K, Tian R, Zhang W, Li Y, Zeng N, Liang Y (2022). alpha-Enolase inhibits apoptosis and promotes cell invasion and proliferation of skin cutaneous melanoma. Mol Biol Rep.

[50] Fu QF, Liu Y, Fan Y, Hua SN, Qu HY, Dong SW (2015). Alpha-enolase promotes cell glycolysis, growth, migration, and invasion in non-small cell lung cancer through FAK-mediated PI3K/AKT pathway. J Hematol Oncol.

[51] Ji M, Wang Z, Chen J, Gu L, Chen M, Ding Y (2019). Up-regulated ENO1 promotes the bladder cancer cell growth and proliferation via regulating beta-catenin. Biosci Rep.

[52] Shi X, Jin L, Dang E, Chang T, Feng Z, Liu Y (2011). IL-17A upregulates keratin 17 expression in keratinocytes through STAT1- and STAT3-dependent mechanisms. J Invest Dermatol.

[53] Yang L, Jin L, Ke Y, Fan X, Zhang T, Zhang C (2018). E3 Ligase Trim21 Ubiquitylates and Stabilizes Keratin 17 to Induce STAT3 Activation in Psoriasis. J Invest Dermatol.

[54] Bai X, Yu C, Yang L, Luo Y, Zhi D, Wang G (2020). Anti-psoriatic properties of paeoniflorin: suppression of the NF-kappaB pathway and Keratin 17. Eur J Dermatol.

[55] Henriet E, Abdallah F, Laurent Y, Guimpied C, Clement E, Simon M (2023). Targeting TGF-beta1/miR-21 Pathway in Keratinocytes Reveals Protective Effects of Silymarin on Imiquimod-Induced Psoriasis Mouse Model. JID Innov.

[56] Makuch S, Kupczyk P, Makarec A, Chodaczek G, Ziolkowski P, Wozniak M (2023). The Impact of Proinflammatory Cytokines and Imiquimod on GLUT1 in HaCaT Keratinocytes - a Potential Anti-Psoriatic Therapeutic Target?. Cell Physiol Biochem.

[57] Sutter CH, Olesen KM, Bhuju J, Guo Z, Sutter TR (2019). AHR Regulates Metabolic Reprogramming to Promote SIRT1-Dependent Keratinocyte Differentiation. J Invest Dermatol.

[58] Tohgasaki T, Ozawa N, Yoshino T, Ishiwatari S, Matsukuma S, Yanagi S (2018). Enolase-1 expression in the stratum corneum is elevated with parakeratosis of atopic dermatitis and disrupts the cellular tight junction barrier in keratinocytes. Int J Cosmet Sci.

[59] Chung BM, Arutyunov A, Ilagan E, Yao N, Wills-Karp M, Coulombe PA (2015). Regulation of C-X-C chemokine gene expression by keratin 17 and hnRNP K in skin tumor keratinocytes. J Cell Biol.

