# Supplementary material for: Keratin 17 covalently binds to alpha-enolase and exacerbates proliferation of keratinocytes in psoriasis
Source: Int J Biol Sci. 2023 Jul 3;19(11):3395–411. doi: 10.7150/ijbs.83141 (PMC10367554; doi:10.7150/ijbs.83141)
Supplement: Supplementary file 1 — Supplementary figures and tables. [file ijbsv19p3395s1.pdf]

ONLINE REPOSITORY MATERIALS

Supplementary Tables

Table S1. The primer sequences used in the ChIP assay

**Primer name**		**sequence 5′→3′**
LDHA segment 1	Forward	GTGGTCACATTTGGTAGGCA
	Reverse	AAATGGGGCTCTCACCTCAAA
LDHA segment 2	Forward	GGCATACTCAAGGGCTTACCA
	Reverse	TGATTCCATTGCCTAGCCCG
LDHA segment 4	Forward	CAGTGGGGTGGGCAGTAGA
	Reverse	GTGACCTCCAAAACTCGACG
LDHA segment 5	Forward	CCTCCCCAGGTTTCATGGAT
	Reverse	CTAAGTAAGGTGCTGCCCTCC

Table S2. siRNA sequences

Gene symbol		siRNA Sequences
Human sense K17 siRNA	Sense	CCTGACTCAGTACAAGAAATT
	Anti-sense	UUUCUUGUACUGAGUCAGGTT
Human sense ENO1 siRNA	Sense	GCAUUGGAGCAGAGGUUUAdTdT
	Anti-sense	CCTGACTCAGTACAAGAAATT
Control sense siRNA	Sense	UUCUCCGAACGUGUCACGUTT
	Anti-sense	ACGUGACACGUUCGGAGAATT

Table S3. Primers for the analysis of mRNAs

**Primer name**		**sequence 5′→3′**
Human *K17*	Forward	CCAGCTCAGCATGAAAGCATC
	Reverse	ACCTCTTCCACAATGGTACGC
Human *K16*	Forward	GACCGGCGGAGATGTGAAC
	Reverse	CTGCTCGTACTGGTCACGC
Human *GLUT1*	Forward	TCTGGCATCAACGCTGTCTTC
	Reverse	CGATACCGGAGCCAATGGT
Human *PKM2*	Forward	AAGGGTGTGAACCTTCCTGG
	Reverse	GCTCGACCCCAAACTTCAGA
Human *PGK1*	Forward	GACCTAATGTCCAAAGCTGAGAA
	Reverse	CAGCAGGTATGCCAGAAGCC
Human *PFKM*	Forward	GGAAAGGAAGACAGAGTGGGAGGC
	Reverse	CAGATCGACCTCAACAGTGGGATTC
Human *ENO1*	Forward	GGAAAGGAAGACAGAGTGGGAGGC
	Reverse	CAGATCGACCTCAACAGTGGGATTC
Human *HK2*	Forward	TGATCGCCTGCTTATTCACGG
	Reverse	AACCGCCTAGAAATCTCCAGA
Human *LDHA*	Forward	CATTGTCAAGTACAGTCCACACT
	Reverse	TTCCAATTA CTCGGTTTTTGGGA
Human *PCNA*	Forward	GCGTGAACCTCACCAGTATGT
	Reverse	TCTTCGGCCCTTAGTGTAATGAT

Human *Cyclin D1*	Forward	TGGAGCCCGTGAAAAAGAGC
	Reverse	TCTCCTTCATCTTAGAGGCCAC
Human *β-Actin*	Forward	GGCTACAGCTTCACCACCAC
	Reverse	TGCGCTCAGGAGGAGC
Mouse *K17*	Forward	GCCCACCTGACTCAGTACAA
	Reverse	GGAGCTGAGTCCTTAACGGG
Mouse *K16*	Forward	ATGCACAGTTCACTTTGCAGA
	Reverse	CGCAAGAACAGCTCATTCTCG
Mouse *GLUT1*	Forward	GCAGTTCGGCTATAACACTGG
	Reverse	GCGGTGGTTCCATGTTTGATTG
Mouse *ENO1*	Forward	TGTGGCTGCCTCCGAGTTCTAC
	Reverse	ACTGGGTAGTTCTGGACGAAGGAC
Mouse *PGK1*	Forward	CAAATTCTGCTTGGACAATGGA
	Reverse	CCACACAATCCTTCAAGAACAG
Mouse *PKM2*	Forward	TATCATTGCCGTGACTCGAAAT
	Reverse	AAGTTTACACGAAGGTCGACAT
Mouse *HK2*	Forward	CTACATGGAGGAGATGCGTAAT
	Reverse	GCTTTGTGAAATCGATCAGGAT
Mouse *LDHA*	Forward	AAGACTACTGTGTAACTGCGAA
	Reverse	ACTTGAAGATGTTCACGTTTCG
Mouse *PCNA*	Forward	TTTGAGGCACGCCTGATCC
	Reverse	GGAGACGTGAGACGAGTCCAT

Mouse *Cyclin D1*	Forward	GCGTACCCTGACACCAATCTC
	Reverse	ACTTGAAGTAAGATACGGAGGGC
Mouse *β-Actin*	Forward	AGCCTTCCTTCTTGGGTATG
	Reverse	GCTCAGTAACAGTCCGCCTA

Supplementary Figures

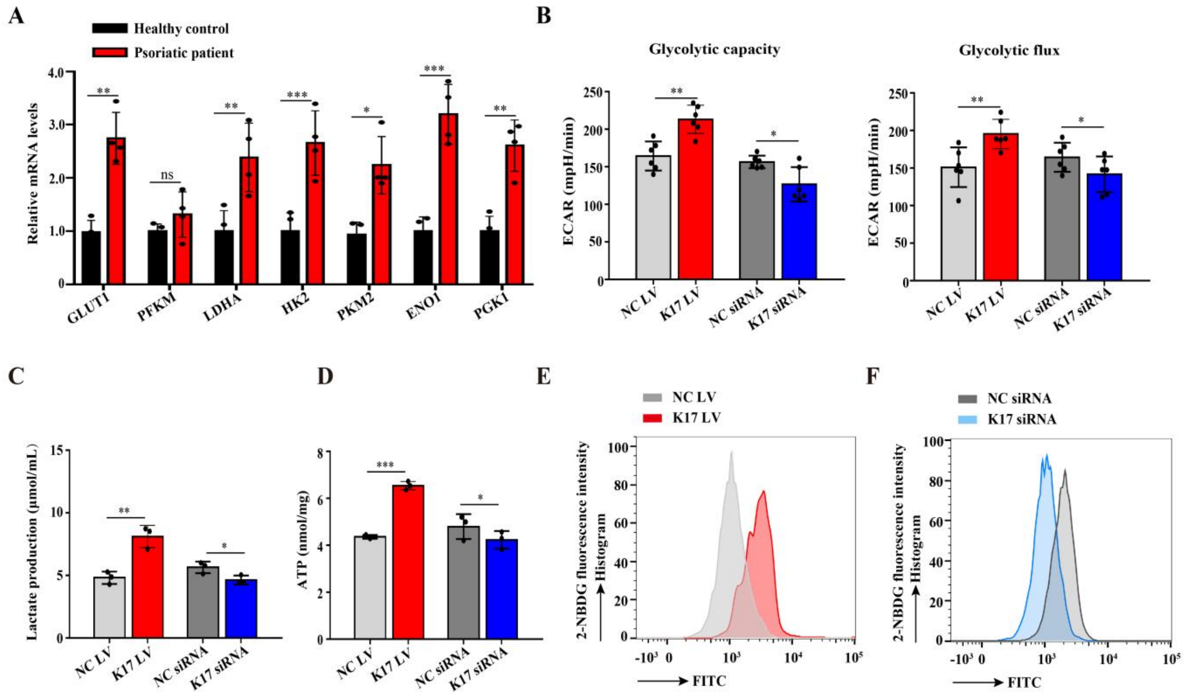

Supplementary Figure 1. Levels of key genes associated with glycolysis in psoriasis and the effect of K17 expression on glycolysis in KCs. (**A**) mRNA expression of the key genes associated with glycolysis in the lesional skin of psoriasis patients (*n* = 4) and healthy controls (*n* = 4). (**B**) The extracellular acid ratio (ECAR), extracellular lactate production (**C**), intracellular ATP levels (**D**) and the uptake of glucose (**E, F**) were analyzed in KCs with K17 overexpression or K17 siRNA transfection. (*n* = 5 for each group). Data are presented as the mean ± SEM *(n* = 3-5*)*. **p*<0.05, ***p*<0.01, ****p*<0.001, ns, not significant. *p* values were calculated by unpaired Student's *t test*. All experiments were repeated at least three times.

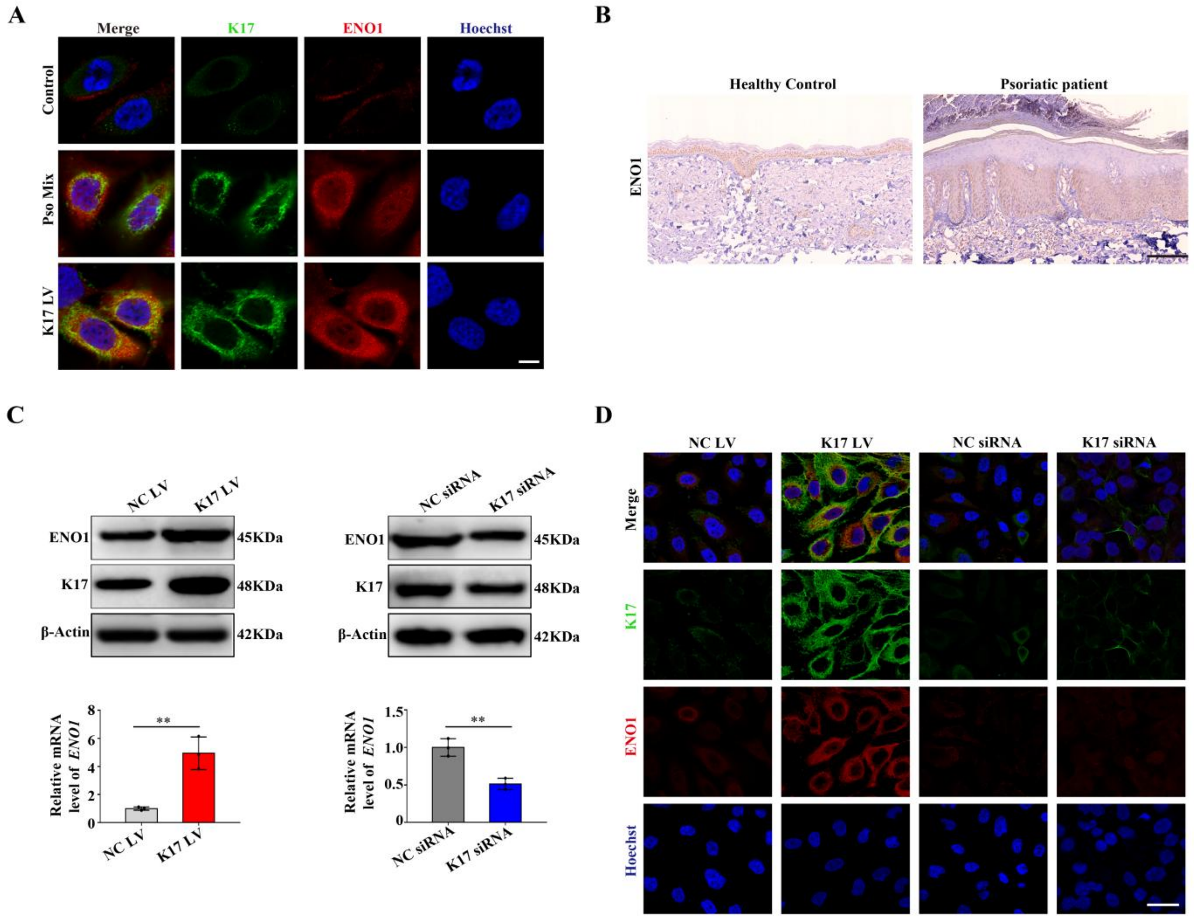

Supplementary Figure 2. Expression of ENO1 and its relationship with K17. (**A**) Immunofluorescence colocalization staining for K17 (green), ENO1 (red) and Hoechst (blue) in KCs stably overexpressing K17 and Pso-Mixed-treated KCs. Scale bar, 10 μm. (**B**) Representative IHC staining of ENO1 in the lesional skin of psoriasis patients (n = 3) and healthy controls (n = 3). Scale bar, 100 μm. (**C**) ENO1 protein and mRNA levels in K17-overexpressing KCs and K17 siRNA-transfected KCs were determined using Western blotting and RT–qPCR. (**D**) Immunofluorescence for ENO1 (red), K17 (green) and Hoechst (blue) in Pso-Mixed KCs, K17-overexpressing KCs and K17 siRNA-transfected KCs. Scale bar, 20 μm. Data are the mean ± SEM *(n* = 3-5*)*. **p<0.01, p values were calculated by unpaired Student's *t test*. All experiments were repeated at least three times.

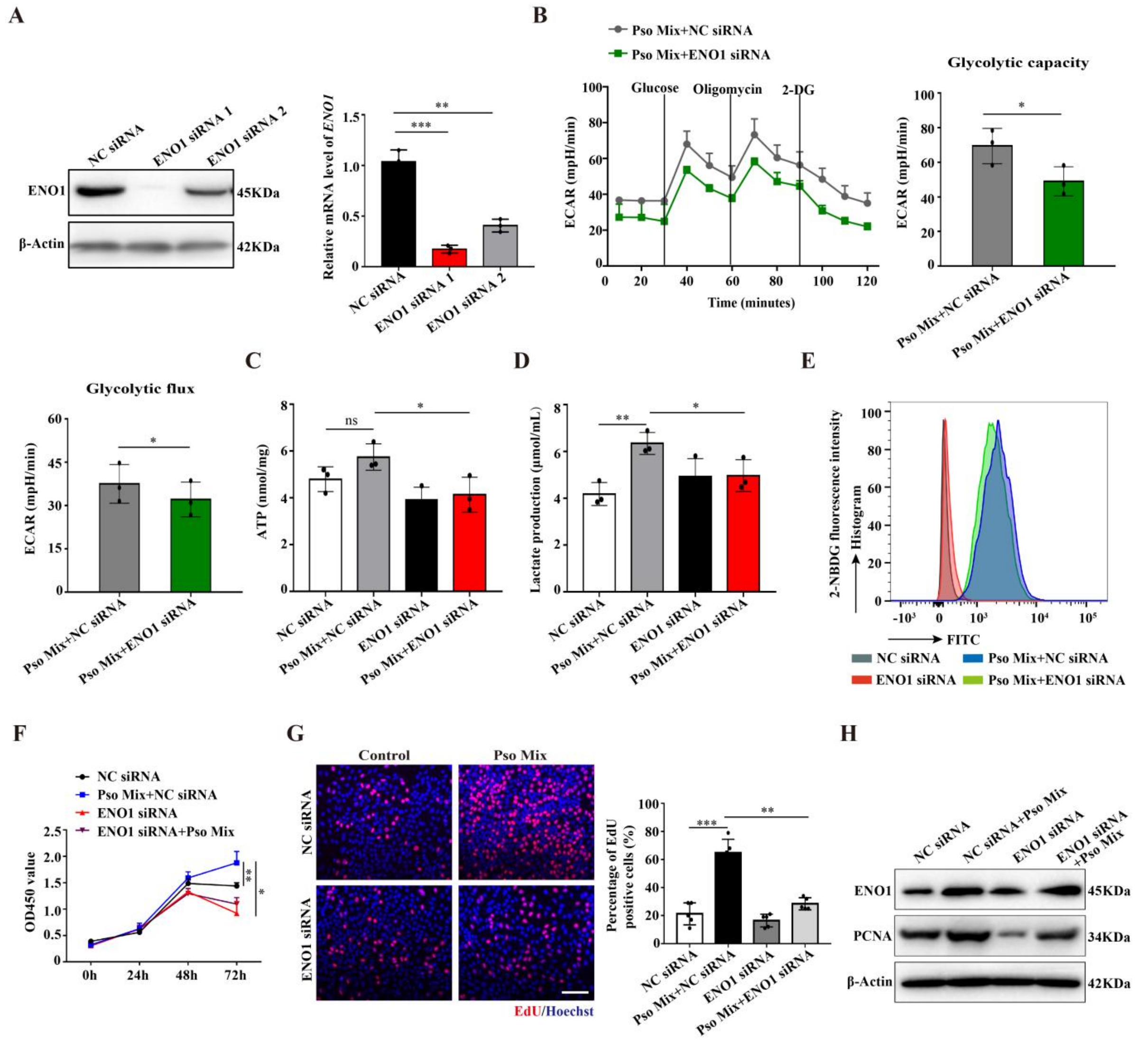

Supplementary Figure 3. Loss of ENO1 inhibits glycolysis and proliferation of psoriatic KCs. (**A**) The efficiency of ENO1 siRNA in KCs was determined using Western blotting and RT–qPCR. (**B**) The extracellular acid ratio (ECAR), intracellular ATP levels (**C**) extracellular lactate production (**D**) and uptake of glucose (**E**) were analyzed in ENO1 siRNA-transfected KCs (*n* = 3-5 for each group). (**F**) Cell proliferation was analyzed by Cell Counting Kit-8 (CCK8) assay, EdU assay, the percentage of EdU positive cells (**G**) and PCNA expression (**H**) in ENO1 siRNA-transfected KCs. Data are the mean ± SEM *(n* = 3-5*)*. **p*<0.05, ***p*<0.01, ****p*<0.001. *p* values were calculated by unpaired Student's *t test*. All experiments were repeated at least three times.

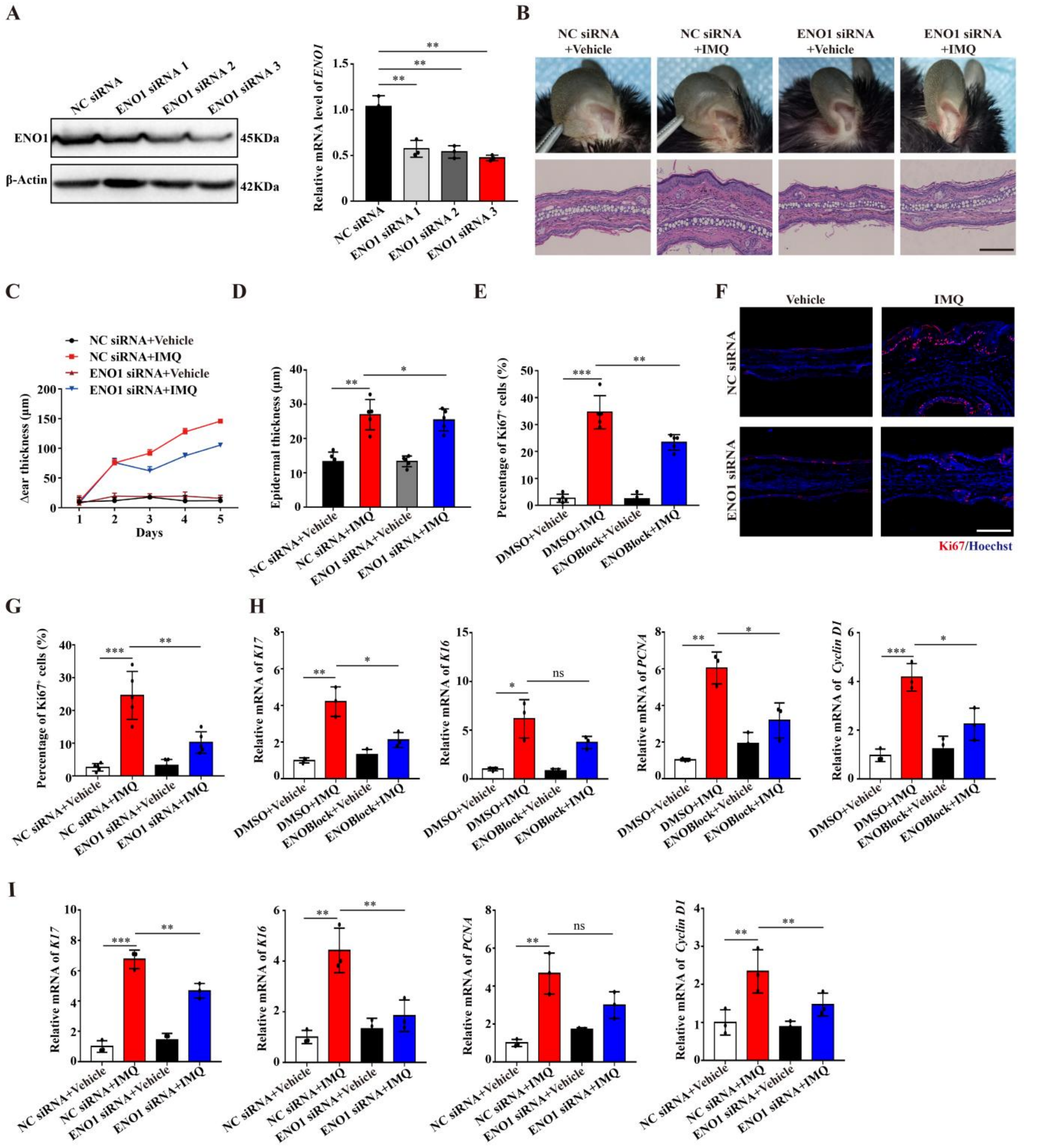

Supplementary Figure 4. ENO1 siRNA significantly attenuates IMQ-induced psoriasis-like inflammation. Local application of ENO1 siRNA once a day in IMQ-induced psoriasis-like mice. NC siRNA was used as a control. (**A**) Validation of mouse ENO1 siRNA interference efficiency in a mouse hepatocellular carcinoma cell line (Hepa 1-6) using Western blotting and RT–qPCR. (**B**) Ear phenotype and H&E staining of lesional skin sections. Scale bar, 10 μm. (**C**) Examination of Δear thickness, *n* = 3 per group. (**D**)

Examination of epidermal thickness, *n* = 3 per group. (**E**) The percentage of $Ki67^+$ cells in the epidermis from ENO1 inhibitor ENOBlock-treated IMQ-induced psoriasis-like mice. (**F**) Immunofluorescence staining of Ki67 and (**G**) the percentage of $Ki67^+$ cells in the epidermis from ENO1 siRNA-treated IMQ-induced psoriasis-like mice. Ki67 (red) and Hoechst (blue). Scale bar, 50 μm. (**H**) Relative mRNA expression of *K17*, *K16*, *PCNA*, and *Cyclin D1* in IMQ-induced psoriasis-like inflammation in ENOBlock-treated mice. (**I**) Relative mRNA expression of *K17*, *K16*, *PCNA*, and *Cyclin D1* in IMQ-induced psoriasis-like inflammation in ENO1 siRNA-treated mice. Data are the mean ± SEM *(n* = 3-5*)*. *$p<0.05$, **$p<0.01$, ***$p<0.001$, ns, not significant. *p* values were calculated by unpaired Student's *t test*. All experiments were repeated at least three times.

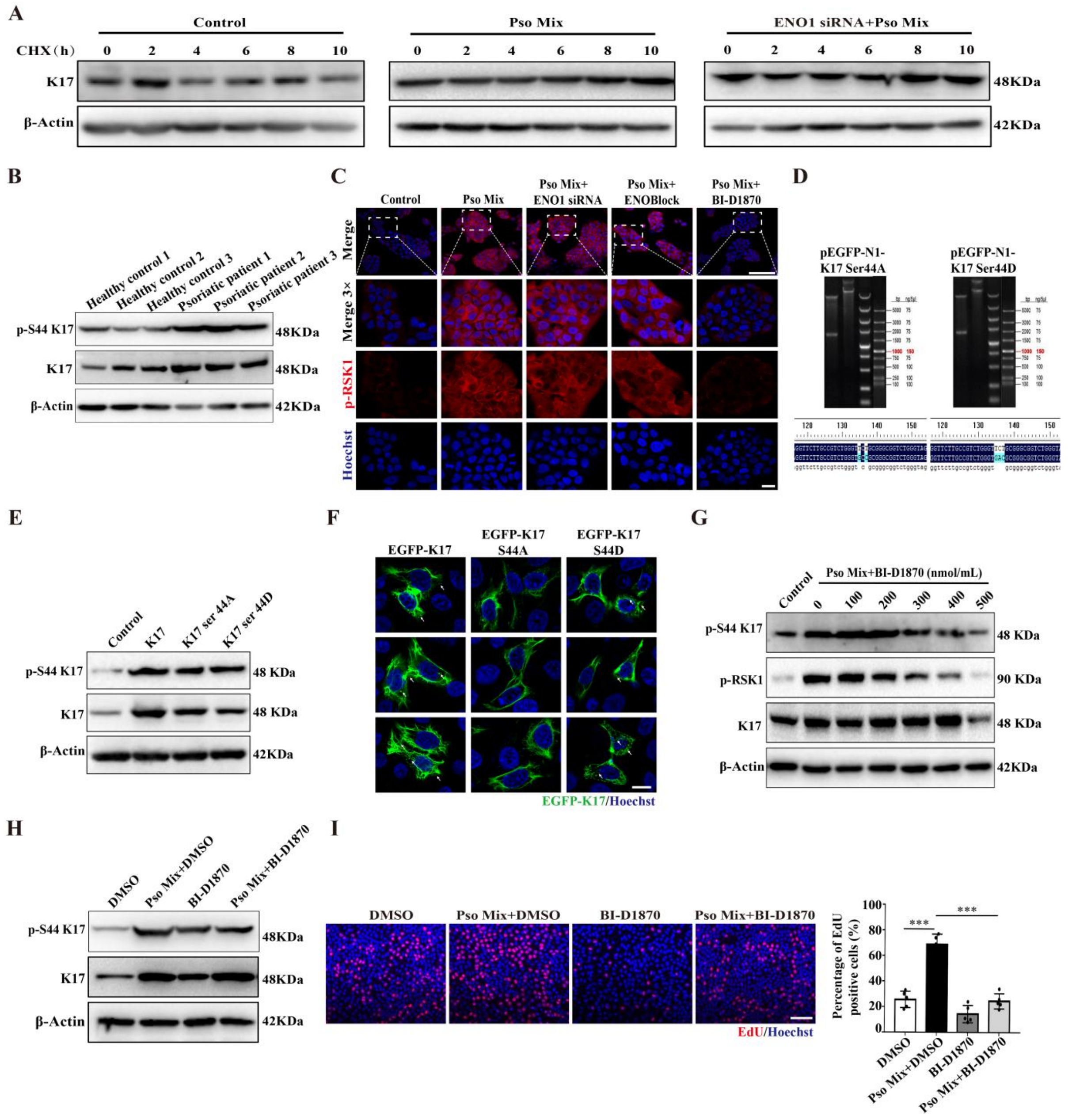

Supplementary Figure 5. ENO1 promotes cell glycolysis and proliferation by regulating the phosphorylation of K17-Ser^{44} (**A**) Immunoblotting and quantitative analysis of K17 protein levels in Con, Pso Mix or ENO1 siRNA-transfected and Pso Mix-treated KCs treated with cycloheximide (CHX, 50 μg/mL). (**B**) Phosphorylation of K17-Ser^{44} protein levels in psoriatic lesional skin (n = 3) and healthy skin (n = 3). (**C**) Immunofluorescence staining for p-RSK1 (red) and Hoechst (blue) in KCs treated with Pso Mix and ENO1 siRNA-transfected, ENOBlock-treated or BI-D1870-treated KCs. Scale bar, 50 μm (upper)/10 μm (lower). (**D**) Construction of the K17 mutant overexpression plasmids pEGFP-N1-K17 S44A and

pEGFP-N1-K17 S44D. (**E**) Phosphorylation of K17-Ser^{44} protein levels in pEGFP-N1-K17-, pEGFP-N1-K17 S44A-, and pEGFP-N1-K17 S44D-transfected KCs. (**F**) Immunofluorescence staining for EGFP-K17 (green), EGFP-K17 S44A (green) and EGFP-K17 S44D (green). White arrows: the punctate and diffuse forms of EGFP-K17. Scale bar, 10 μm. (**G**) The efficiency of different concentrations of BI-D1870. (**H**) Phosphorylation of K17-Ser^{44} protein in BI-D1870-treated KCs. (**I**) Cell proliferation and the percentage of EdU positive cells in BI-D1870-treated KCs were analyzed by EdU assay. Data are the mean ± SEM *(n* = 3-5*)*. ***p<0.001. p values were calculated by unpaired Student's *t test*. All experiments were repeated at least three times.

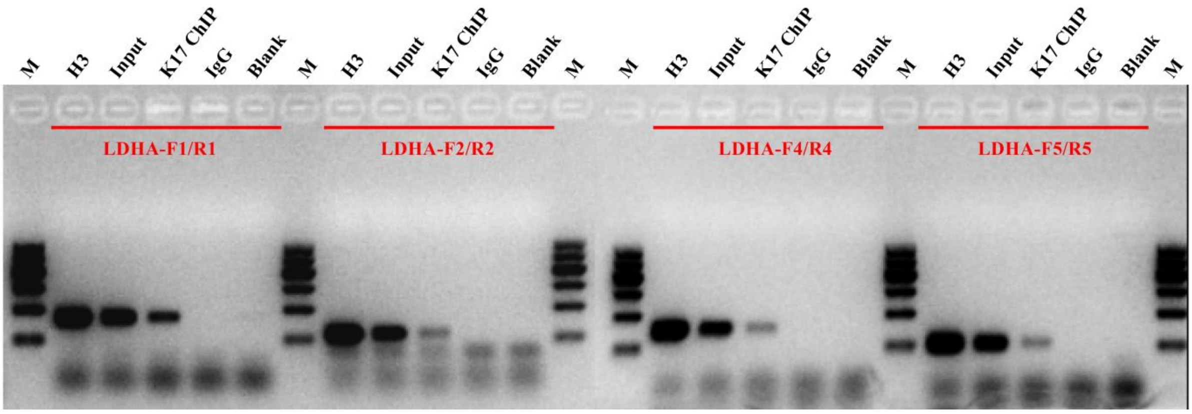

Supplementary Figure 6. Detection of the binding of K17 to the LDHA promoter region. The binding ability of K17 to LDHA promoter DNA was detected by 2% agarose gel electrophoresis. H3: Positive control, histone H3 (D2B12) XP rabbit mAb (#4620); IgG: Negative control, normal rabbit IgG antibody (#2729). All experiments were repeated at least three times.

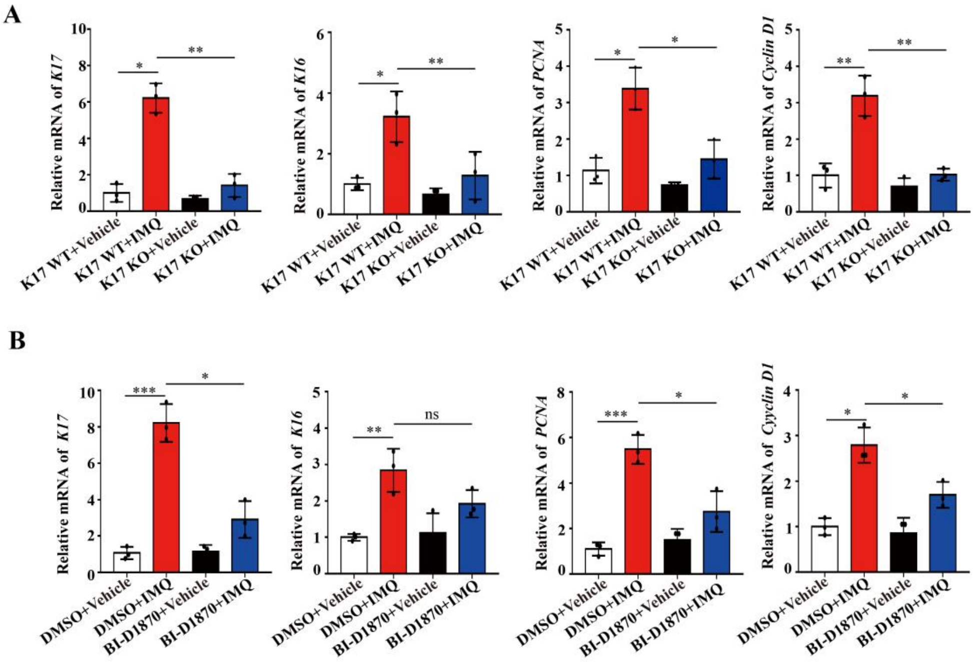

Supplementary Figure 7. Levels of key genes associated with proliferation in IMQ-induced psoriasis-like mice. (**A**) Relative mRNA expression of *K17*, *K16*, *PCNA*, and *Cyclin D1* in IMQ-induced psoriasis-like inflammation in K17 KO or K17 WT mice. (**B**) Relative mRNA expression of *K17*, *K16*, *PCNA*, and *Cyclin D1* in IMQ-induced psoriasis-like inflammation in BI-D1870-treated mice. Data are presented as the mean ± SEM *(n* = 3-5*)*. **p*<0.05, ***p*<0.01, ****p*<0.001, ns, not significant. *p* values were calculated by unpaired Student's *t test*. All experiments were repeated at least three times.
